# Cognitive-attitudinal factors predict CBT-I enrollment willingness in Chinese sleep clinic patients: a knowledge-attitudes-practices survey

**DOI:** 10.3389/fpsyt.2026.1804213

**Published:** 2026-06-22

**Authors:** Chenglin Zou, Yanping Lan, Xiaofei Liu, Xingzhong Zhu

**Affiliations:** 1 Department of Psychiatry, Ganzhou Hospital-Nanfang Hospital, Southern Medical University (Ganzhou People’s Hospital), Ganzhou, Jiangxi, China; 2 Department of Respiratory and Critical Care Medicine, Ganzhou Hospital-Nanfang Hospital, Southern Medical University (Ganzhou People’s Hospital), Ganzhou, Jiangxi, China; 3 Department of Rehabilitation Medicine, Ganzhou Hospital-Nanfang Hospital, Southern Medical University (Ganzhou People’s Hospital), Ganzhou, Jiangxi, China

**Keywords:** cognitive behavioral therapy, health knowledge attitudes practice, insomnia, mental health services, patient acceptance of health care, sleep disorders

## Abstract

**Background:**

Despite strong evidence for cognitive behavioral therapy for insomnia (CBT-I), uptake remains constrained by poorly understood cognitive, attitudinal, and practical barriers. This study examined determinants of willingness to enroll in sleep improvement programs among adults at risk of sleep disorders—including insomnia, obstructive sleep apnea, and comorbid psychological distress—attending a tertiary sleep clinic in China.

**Methods:**

A cross-sectional knowledge-attitudes-practices survey was conducted among 2,661 adults attending the sleep and behavioral medicine outpatient clinic at Ganzhou Hospital-Nanfang Hospital, Southern Medical University, Ganzhou, Jiangxi, China, between February 2022 and June 2025. Willingness to enroll in a structured sleep improvement program was assessed alongside sleep health knowledge, perceived need, CBT-I versus medication effectiveness beliefs, telehealth acceptability, clinical severity (Insomnia Severity Index, Epworth Sleepiness Scale, STOP-Bang), psychological symptoms (PHQ-2, GAD-2), perceived barriers, and sociodemographic characteristics. Multivariable logistic regression identified independent predictors of willingness to enrollment, with secondary analyses evaluating model discrimination and testing prespecified interactions.

**Results:**

Among 2,661 participants (median age 45 years, 56.5% female, median ISI = 13), 1,386 (52.1%) expressed willingness to enroll. Univariable comparisons showed no significant differences between willing and not-willing groups across demographics, clinical characteristics, or barriers (all p>0.05). However, multivariable modeling revealed that when considered simultaneously, cognitive-attitudinal factors emerged as significant independent predictors, suggesting complex interactions rather than simple bivariate associations. In multivariable models, perceived need (OR = 1.20, 95% CI: 1.16–1.25, p<0.001), beliefs that CBT-I is more effective and durable than sleep medication (OR = 1.12, 95% CI: 1.08–1.16, p<0.001), sleep health and treatment knowledge (assessed by a six-item knowledge score) (OR = 1.09, 95% CI: 1.05–1.13, p<0.001), and anxiety symptoms (OR = 1.07, p=0.005) positively predicted willingness. Paradoxically, depression symptoms (OR = 0.94, p<0.001) and insomnia severity (OR = 0.93, p<0.001) inversely predicted willingness. Model discrimination was modest (AUC = 0.543, 95% CI: 0.504–0.590). Time (mean 3.54) and cost (3.44) were most severe barriers but showed no independent association with willingness (p>0.05).

**Conclusion:**

Cognitive-attitudinal factors (perceived need, CBT-I beliefs, knowledge) independently predicted enrollment willingness, whereas demographics and practical barriers did not. Depression and insomnia severity paradoxically reduced willingness, creating an inverse care law. However, poor model discrimination and measurement of stated willingness rather than actual enrollment limit conclusions. Prospective validation and motivational enhancement strategies for patients with depression are needed.

## 1 Introduction

Sleep health is a global public health priority because insufficient sleep and untreated sleep disorders impair cognition, cardiometabolic health, mental well-being, and safety across the life course (1, 2). The burden remains substantial: modelling suggests ~936 million adults have obstructive sleep apnea (OSA) worldwide, most undiagnosed (3, 4). Sleep disturbance and common mental disorders are tightly linked; insomnia elevates subsequent risk of depression and anxiety two- to three-fold, and sleep-focused interventions reduce depressive and anxiety symptoms (5, 6). During infectious disease epidemics, pooled estimates show high co-occurrence of depression, anxiety, and insomnia, particularly among high-risk groups (6, 7). Together, these data underscore sleep assessment and treatment as core components of population mental-health strategies.

Despite strong evidence for effective care, implementation lags. Cognitive behavioral therapy for insomnia (CBT-I) is first-line and produces durable clinical benefits (8, 9); it can be delivered in person or via telehealth and adapted across settings (10). Yet uptake is constrained by low public literacy about behavioral treatments, limited clinician training and referral pathways, costs and reimbursement, and service capacity (11, 12). Many people regard insomnia as benign or purely psychological and do not seek care; those who do often confront time and logistical barriers (13–15). Understanding how knowledge, attitudes, and practical constraints shape willingness to engage with sleep-improvement programs is therefore essential.

Digital delivery has expanded access where specialist services are scarce. Randomized trials and meta-analyses indicate that internet- or app-based CBT-I yields moderate improvements in insomnia severity and sleep quality with parallel gains in mood and functioning (13, 16). However, engagement depends on perceptions of treatment effectiveness, telehealth acceptability, and feasibility (time, cost, insurance), which vary by sociodemographic context and health-system design (6, 17). Identifying which perceptions and barriers most influence participation is key to designing scalable, equitable interventions (6).

China is a critical setting for this inquiry. With rapid ageing and urbanization, national and regional surveys report high prevalence of sleep problems; a recent multi-province study estimated that about one in five adults meet criteria for a clinically significant sleep disorder, with heterogeneity by age, sex, and socioeconomic position (6, 14). Insomnia symptoms are common in older adults and predict adverse outcomes, emphasizing the need for early, non-pharmacologic management (18, 19). Health-insurance expansion through Urban Employee Basic Medical Insurance (UEBMI) and Urban–Rural Resident Basic Medical Insurance (URRBMI) has improved coverage, but out-of-pocket costs, fragmented care pathways, and variable reimbursement for behavioral therapies persist. Stigma and low perceived need also remain important deterrents to professional help-seeking, especially outside major cities (20).

At the same time, China is a testbed for digital health. A pilot randomized trial of a culturally adapted, smartphone-based CBT-I app showed significant Insomnia Severity Index reductions compared with digital sleep education, supporting local feasibility (13). Yet an age-related digital divide—exacerbated during COVID-19—limits access and comfort with mobile health, e-registration systems, and online payment among older and socioeconomically disadvantaged adults (17, 21). These realities suggest that beliefs about behavioral treatment versus medication, preferences for telehealth, and constraints related to time, cost, and insurance may be decisive determinants of program enrollment in Chinese clinics.

While Knowledge-Attitudes-Practices (KAP) frameworks provide a useful lens, and recent Chinese studies document knowledge gaps and negative attitudes affecting sleep practices (22, 23), a significant evidence gap remains regarding how these factors translate into concrete enrollment decisions for sleep improvement programs. This gap is twofold. Internationally, there is limited quantitative evidence connecting KAP constructs, perceived need, and treatment beliefs to real-world enrollment willingness, as studies often isolate single determinants or focus on intention rather than proximal decisions, seldom integrating clinical severity and practical constraints (11, 17, 24). Specifically, within China, despite high documented need and barriers, little work has holistically evaluated how these factors—combined with beliefs about behavioral versus pharmacologic care (e.g., CBT-I vs. medication), telehealth acceptability, trust in specialists, clinical severity, and mental health symptoms—shape enrollment decisions in hospital-based clinics (1, 22, 23). Moreover, potential moderation of key associations by factors like sex or psychological distress is also largely untested (21, 24).

To address these gaps, we conducted a single-center cross-sectional KAP survey in a Chinese hospital outpatient clinic. We quantified adults’ willingness to enroll in a structured sleep-improvement program and examined its independent determinants using multivariable modelling that incorporated knowledge about sleep and treatments, perceived need, comparative beliefs about CBT-I versus medication, telehealth acceptability, trust in a sleep specialist, clinical severity indices (Insomnia Severity Index, Epworth Sleepiness Scale, STOP-Bang), psychological symptoms (PHQ-2, GAD-2), and perceived barriers (time, cost, insurance, stigma, digital access), alongside sociodemographic and risk-flag covariates. Secondary aims were to evaluate model discrimination and calibration and to test two prespecified interactions—knowledge with sex and perceived need with psychological distress—to inform targeting and delivery strategies.

## 2 Methods

### 2.1 Study design and setting

We conducted a single-center, cross-sectional KAP survey at a tertiary sleep and behavioral medicine clinic in Ganzhou, China, between February 2022 and June 2025. The investigation evaluated determinants of willingness to enroll in a structured sleep-improvement program offered in routine care. The program, consistent with guideline-concordant cognitive behavioral therapy for insomnia (CBT-I), was available via in-person group sessions or synchronous telehealth, subject to service capacity. The study adhered to a prespecified analysis plan and conforms to STROBE recommendations for observational studies.

### 2.2 Participants and recruitment

Adults (≥18 years) presenting for new or return visits to the sleep/behavioral-medicine clinic were screened consecutively by trained research staff during clinic hours. Eligibility criteria included: (i) self-reported sleep complaint or risk factors for sleep disturbance (e.g., snoring, witnessed apnea, insomnia symptoms, non-restorative sleep, daytime sleepiness), or referral for sleep evaluation; (ii) capacity to provide informed consent and complete a brief questionnaire in Mandarin; and (iii) residence in the catchment area during the study period. Exclusion criteria were: (i) acute medical or psychiatric instability precluding survey completion; (ii) severe cognitive impairment; and (iii) inability to communicate in Mandarin or with an interpreter. No *a priori* sample size calculation was performed as all eligible attendees during the 3.3-year accrual window were consecutively recruited. A formal screening log recording the number of individuals approached and the specific reasons for each exclusion was not maintained prospectively; this precludes generation of a complete CONSORT-style participant flow diagram and is acknowledged as a study limitation. All 2,661 participants who completed the survey and met eligibility criteria are included in analyses. *Post-hoc* power analysis indicates that with N = 2,661 and outcome prevalence of 52%, the study had >99% power to detect OR≥1.15 for continuous predictors (α=0.05, two-tailed), ensuring adequate precision for the prespecified multivariable models.

### 2.3 Variables and measurements

All variables were operationalized *a priori* and measured using standardized, Mandarin-language instruments administered immediately prior to or following the clinical consultation. Unless otherwise noted, Likert items used a 5-point scale (1=strongly disagree to 5=strongly agree), with higher scores indicating greater endorsement.

#### 2.3.1 Sociodemographic and access characteristics

Age (years) was self-reported and treated as a continuous variable. Sex was recorded as female or male. Education was categorized as primary or below, junior high, senior/vocational, college/university (reference), or postgraduate, aligned with Chinese educational strata. Residency registration (hukou) was classified as local city (reference), non-local in-province, or non-local out-of-province. Health-insurance coverage was recorded as Urban Employee Basic Medical Insurance (UEBMI; reference), Urban–Rural Resident Basic Medical Insurance (URRBMI), commercial insurance, or none. Visit type was recorded as new (reference) or return. Employment schedule captured predominant work timing in the past month (daytime [reference], evening, night, rotating, unemployed, or student). Household size (including the respondent; persons living in the dwelling) and persons per bedroom were self-reported. Travel time to clinic (minutes, one-way, door-to-door, excluding clinic waiting time) was recorded as a continuous measure.

#### 2.3.2 Sleep and clinical measures

Sleep duration on workdays and free days (hours/night) were self-reported using a typical-week frame. Respondents were instructed to report within 0–12 hours; data-entry validation permitted 0–16 hours to flag and review outliers. Sleep latency (minutes) and nocturnal awakenings (number/night) were obtained from patient report. The Insomnia Severity Index (ISI; 7 items, 0–28), Epworth Sleepiness Scale (ESS; 8 items, 0–24), STOP-Bang (8 items, 0–8; OSA risk flag ≥3), PHQ-2 (0–6), and GAD-2 (0–6) were administered in their validated Mandarin versions without modification. Each has established psychometric support in Chinese populations: the ISI demonstrates good internal consistency (Cronbach’s α = 0.74–0.82) and test-retest reliability (r = 0.79–0.85); the ESS shows acceptable consistency (α = 0.78–0.88) and criterion validity against multiple sleep latency testing; the Mandarin STOP-Bang yields sensitivity 88–93% and specificity 45–58% for AHI ≥15 events/hour against polysomnography; and the PHQ-2 (α = 0.75) and GAD-2 (α = 0.79) have established Chinese validity at the standard screening cutoff of ≥3.

For team-constructed KAP items, internal consistency in the current sample was acceptable for the five attitudinal items (perceived need, CBT-I vs medication belief, trust in specialist, telehealth acceptability, privacy concern; α = 0.71, 95% CI: 0.68–0.74) and the five barrier items (time, cost, insurance, stigma, digital access; α = 0.68, 95% CI: 0.65–0.72). The sleep-hygiene adherence item was retained as a single indicator. The six-item knowledge scale was treated as a formative composite (sum of correct responses, 0–6); mean inter-item correlation was 0.19 (range: 0.08–0.31), consistent with acceptable item diversity in a content-sampled battery.

Snoring or witnessed apnea frequency was assessed with a three-level item (never; occasionally=<3 nights/week; frequently=≥3 nights/week). Prior clinician diagnoses (insomnia, obstructive sleep apnea, depression, anxiety) were recorded as yes/no based on self-report of a previous medical diagnosis. Chronic pain condition and cardiometabolic disease were recorded as yes/no based on self-report of a clinician diagnosis; cardiometabolic disease included any of hypertension, diabetes mellitus, dyslipidemia, or ischemic heart disease, as applicable. Perinatal status (pregnant or ≤1 year postpartum) was recorded among eligible respondents.

#### 2.3.3 Knowledge, attitudes, and practices

The KAP framework was operationalized across three domains as follows.

Knowledge domain: Knowledge score (0–6) was computed as the sum of six single-best-answer items assessing sleep health and CBT-I treatment knowledge (1): optimal sleep-duration ranges; (2) consequences of chronic sleep restriction; (3) first-line treatment for chronic insomnia; (4) risks of long-term hypnotic use; (5) core components of CBT-I; and (6) sleep-hygiene principles. Correct responses were coded 1 and incorrect/’don’t know’ responses 0.

Attitudes domain: Five items captured treatment-related beliefs and healthcare orientations: Perceived need for help (1–5) assessed the respondent’s appraisal of needing professional assistance for current sleep problems; comparative treatment belief (CBT-I vs medication effectiveness, 1–5) captured agreement with the statement that CBT-I is more effective and durable than sleep medication for chronic insomnia; Trust in a sleep specialist (1–5) measured confidence in specialist expertise and recommendations; Telehealth acceptability (1–5) assessed willingness to engage in remote CBT-I sessions; and Privacy concern (1–5) measured data-sharing and confidentiality concerns relevant to digital delivery.

Practices domain: Two items captured behavioral patterns relevant to sleep health management: Sleep-hygiene adherence (1–5) captured the frequency with which respondents followed evidence-informed sleep behaviors (e.g., consistent bed/wake times, stimulus control, caffeine/alcohol timing); and prior participation in a sleep program was recorded as a three-category variable (no; yes, completed; yes, not completed) (55–57).

#### 2.3.4 Perceived barriers to participation

Barriers were measured on 5-point Likert scales with higher values indicating greater barrier severity: time constraints, cost concerns, insurance coverage limitations (e.g., lack of reimbursement for behavioral therapies), stigma related to sleep or mental-health care, and digital access (device/Internet access and digital literacy). Items were developed with clinic stakeholder input to reflect local service realities and phrased neutrally to minimize social desirability bias.

Barrier severity ranking. Barriers were ranked by mean severity scores for clinical interpretation, as median values were identical across barriers (all median=3.0). Mean severity rankings were: Time (mean=3.54), Cost (mean=3.44), Insurance (mean=3.16), Stigma (mean=3.13), and Digital access (mean=2.72).

#### 2.3.5 Mental-health symptoms and psychological distress flag

Depressive symptoms were measured using the 2-item Patient Health Questionnaire (PHQ-2; total 0–6) and anxiety symptoms with the 2-item Generalized Anxiety Disorder scale (GAD-2; total 0–6). The psychological distress flag used in interaction analyses was defined *a priori* as PHQ-2 ≥3 and/or GAD-2 ≥3, indicating positive screening for probable depression or anxiety. PHQ-2 and GAD-2 total scores were entered as continuous predictors in multivariable models; the composite flag was used only for moderation testing.

#### 2.3.6 Derived risk flags

Prespecified binary risk flags (0/1) were constructed to align with clinical plausibility and service planning: Shift/Night Work (employment schedule of night or rotating); Caregiver (≥7 hours/week of unpaid caregiving to a dependent adult/child, when reported); Migrant (non-local hukou); Low Socioeconomic Position (education ≤ junior high and insurance of URRBMI or none); Insomnia (ISI ≥15); OSA Risk (STOP-Bang ≥3); Perinatal Status (pregnant or ≤1 year postpartum); Older Age with Multimorbidity (age ≥60 years with ≥2 chronic conditions among insomnia diagnosis, OSA diagnosis, chronic pain, cardiometabolic disease, depression/anxiety); Chronic Pain or Cardiometabolic Disease (either condition present); and Psychological Distress (as defined above). Risk flags were used descriptively and in Model A to benchmark sociodemographic/clinical correlations of willingness.

### 2.4 Outcome definition

The primary outcome was willingness to enroll in the structured sleep-improvement program. Before the willingness item was administered, a trained research staff member read aloud an identical standardized description to every participant, covering: program type (group-based CBT-I); delivery modality (in-person at the clinic or synchronous telehealth); session schedule (once weekly for six to eight weeks, approximately 60–90 minutes per session); therapeutic components (sleep restriction therapy, stimulus control, cognitive restructuring of dysfunctional sleep beliefs, sleep hygiene education, and relapse prevention); and contextual framing (participation offered within routine clinical care subject to capacity, with no obligation to enroll following survey completion).

Willingness was then rated on a 5-point Likert scale (1 = strongly unwilling to 5 = strongly willing) and dichotomized for analysis as willing (≥4) versus not willing (1–3). This threshold was selected on conceptual and pragmatic grounds: a score of 3 denotes ambivalence rather than a positive expression of intent, and ambivalent respondents are operationally indistinguishable from reluctant ones (scores 1–2) because neither group would proactively self-initiate enrollment without additional motivational support. A cut-point of ≥4 is therefore consistent with behavioral intention research conventions requiring a clear affirmative threshold to predict downstream health-seeking behavior. The full distribution of willingness scores (1 through 5) is reported descriptively to provide transparency. However, for descriptive context, prior participation in sleep programs was recorded as a three-category variable (no; yes, completed; yes, not completed).

### 2.5 Data collection procedures

Data were collected via secure tablet-based surveys or paper forms, with immediate review for completeness. Instruments were developed in Mandarin using plain-language phrasing and underwent expert review by sleep clinicians and behavioral-medicine researchers for content validity. Where applicable, items were adapted from established scales; newly developed items (e.g., barriers, telehealth acceptability, comparative beliefs) were interviewer-piloted for clarity and cultural appropriateness. Standardized instructions and visual anchors were provided for Likert items and time/duration fields. Research staff received training in neutral interviewing, confidentiality, and handling of sensitive topics. Completed forms were entered into a REDCap-compatible database with range and logic checks.

### 2.6 Statistical analysis

All analyses were prespecified. Continuous variables were summarized as medians with interquartile ranges (IQR) given their non-Gaussian distributions; categorical variables were summarized as counts and percentages. Group differences by enrollment willingness were tested using the Mann–Whitney U test for continuous variables and χ² tests for categorical variables. Univariable logistic regressions screened candidate predictors of willingness, reporting odds ratios (OR) with 95% confidence intervals (CI).

Two multivariable models were specified. Model A included sociodemographic covariates and derived risk flags to quantify baseline associations. Model B expanded Model A to incorporate knowledge score, perceived need, comparative CBT-I vs medication belief, trust in a sleep specialist, telehealth acceptability, PHQ-2 and GAD-2 scores, ISI, ESS, STOP-Bang, travel time (log-transformed as log[1+minutes] to reduce skew), and the five barrier scores (time, cost, insurance, stigma, digital). Education, hukou, insurance, visit type, employment schedule, sex, and prior participation were included as categorical covariates with prespecified reference levels matching those used in descriptive tables. Reference categories were prespecified to match analytical needs; any deviations from conventional choices are noted in individual tables. Collinearity was assessed by variance inflation factors; model fit and specification were evaluated using the Hosmer–Lemeshow test and calibration plots.

Exploratory dimensionality reduction. Principal component analysis (PCA) was applied to the full set of KAP-related continuous variables to characterize their latent dimensional structure and empirically evaluate whether cognitive-attitudinal, clinical severity, and barrier items constitute separable domains—a prerequisite for interpreting the relative contributions of these domains in multivariable models. Hierarchical clustering using correlation distance (distance = 1 − |r|) with complete linkage was applied to group variables by their mutual correlation patterns, providing a complementary view of inter-variable architecture. Both analyses were exploratory and secondary to the primary multivariable logistic regression; they were not used to select or exclude predictors from regression models.

**Figure 1:**
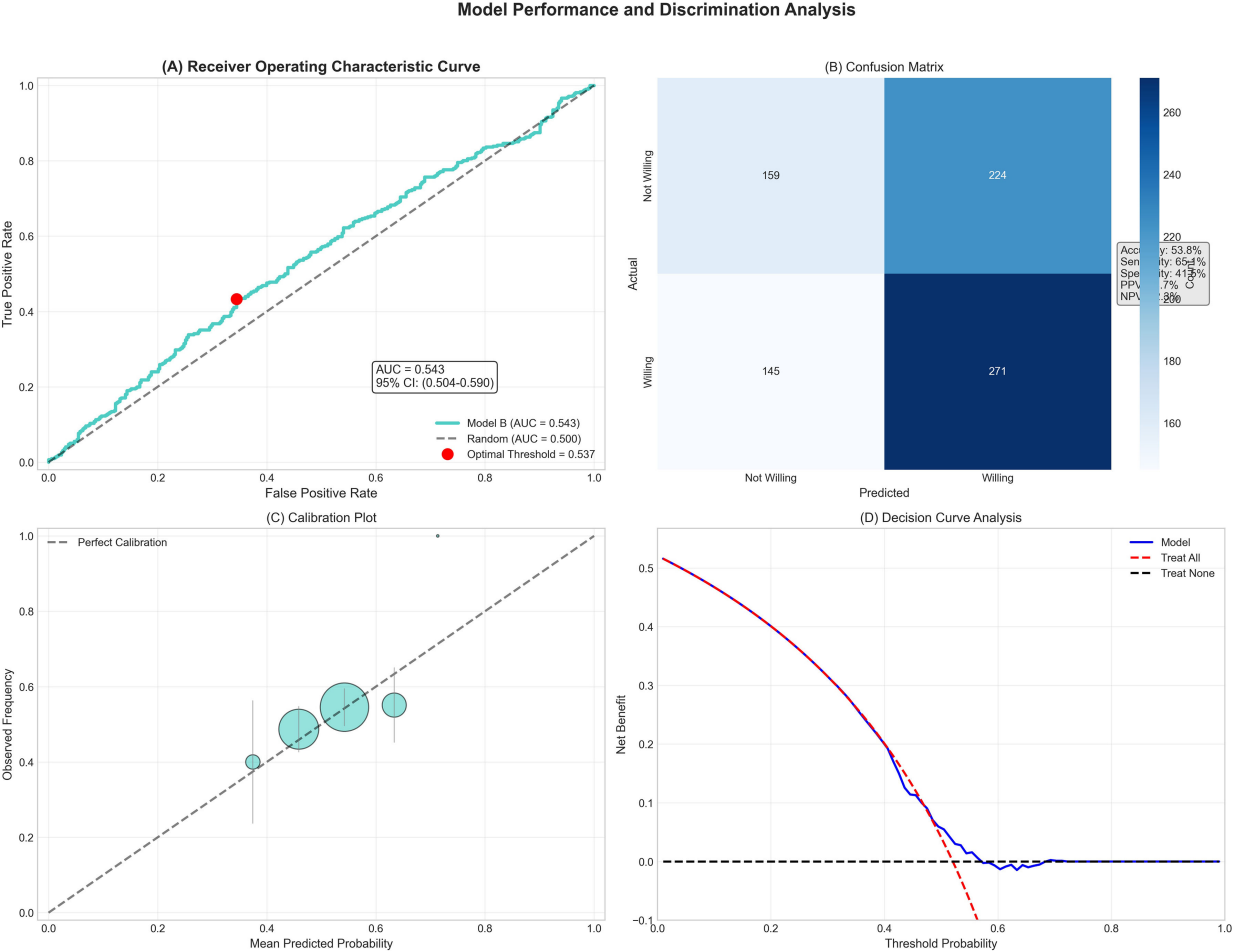
Model performance and discrimination analysis, Final multivariable model demonstrates modest discriminative ability. **(A)** Receiver operating characteristic curve shows area under curve with optimal threshold marked. **(B)** Confusion matrix displays classification performance at optimal threshold. **(C)** Calibration plot demonstrates agreement between predicted probabilities and observed frequencies across risk deciles. **(D)** Decision curve analysis shows minimal net benefit of model-guided decisions compared to treat-all or treat-none strategies.

Model performance was summarized using the area under the receiver operating characteristic curve (AUC) with 95% CI estimated by 2000 bootstrap resamples. The ROC curve plots the true-positive rate (sensitivity) against the false-positive rate (1 − specificity) across all possible classification thresholds; the AUC quantifies overall discriminative ability on a scale from 0.5 (performance equivalent to random chance) to 1.0 (perfect discrimination). The optimal classification threshold was identified using the Youden index (maximizing sensitivity + specificity − 1). At this optimal threshold, overall correct classification rate (accuracy), sensitivity, specificity, positive predictive value (PPV), and negative predictive value (NPV) are reported in the Results and displayed in a confusion matrix (**Figure 1B**). Performance metrics were evaluated on a validation subset (n=799) obtained via a stratified 70/30 train–test split preserving the outcome proportion. Calibration was assessed graphically by plotting observed vs predicted probabilities across deciles of risk with an overlaid loess smoother. Clinical utility was explored using decision-curve analysis (DCA) across a plausible range of threshold probabilities for enrollment outreach. Two interaction terms were tested *a priori*: knowledge score × sex and perceived need × psychological distress flag. These interactions were selected for clinical relevance: the former to probe potential gender differences in health literacy, and the latter to evaluate whether psychological distress modifies treatment-seeking behavior.

### 2.7 Missing data and sensitivity analyses

Data completeness was checked at the point of entry. For the primary analyses, complete-case estimation was implemented. As a sensitivity approach, if any predictor exhibited >5% missingness, multiple imputation by chained equations (10 datasets) would be performed under missing-at-random assumptions, including all variables from Model B and the outcome in the imputation model; estimates would be combined using Rubin’s rules. Sensitivity analyses also examined alternative operationalization’s for OSA risk (STOP-Bang ≥5) and for insomnia (ISI ≥10) and re-estimated Model B to assess robustness of key associations.

### 2.8 Data management and quality assurance

Data were stored on secure institutional servers with access restricted to authorized personnel. Predefined range checks (e.g., sleep duration 0–16 hours; sleep latency 0–240 minutes; travel time 0–300 minutes) and cross-field logic checks (e.g., persons per bedroom ≤ household size; employment schedule mutually exclusive) were executed at entry. An audit trail tracked edits. Interim quality reviews compared paper forms to electronic records for a random 5% sample; discrepancies triggered corrective training. Scale scoring algorithms (ISI, ESS, STOP-Bang, PHQ-2, GAD-2) were implemented in reproducible code with unit tests to prevent scoring errors.

### 2.9 Reporting

Primary and secondary results are reported as adjusted ORs with 95% CIs and two-sided p-values; statistical significance was defined as p<0.05 without multiplicity correction, given the confirmatory focus on prespecified predictors. Descriptive tables, univariable models, multivariable Models A and B, and interaction analyses correspond to **Tables 1**–**8**, and figures include ROC/AUC, calibration, decision-curve, and multivariable effect visualizations, as presented elsewhere in the manuscript.

**Table 1:** Baseline characteristics by willingness to enroll in a sleep-improvement program.

Characteristic	Total (N = 2661)	Not willing (n=1275)	Willing (n=1386)	P value
Demographics				
Age, y	45.0 (35.0–63.0)	45.0 (35.0–62.0)	45.0 (35.0–63.0)	0.489
Travel time to clinic, min	35.0 (25.0–47.0)	36.0 (26.0–47.0)	35.0 (25.0–46.0)	0.11
Household size, No.	3.0 (2.0–4.0)	3.0 (2.0–4.0)	3.0 (2.0–4.0)	0.705
Persons per bedroom, No.	2.0 (1.0–2.0)	2.0 (1.0–2.0)	2.0 (1.0–2.0)	0.892
Sociocultural and access				
Sex				0.417
Female	1504 (56.5%)	731 (57.3%)	773 (55.8%)	
Male	1157 (43.5%)	544 (42.7%)	613 (44.2%)	
Education				0.992
Senior/vocational	962 (36.1%)	460 (36.1%)	502 (36.2%)	
College/University	746 (28.0%)	351 (27.5%)	395 (28.5%)	
Junior high	470 (17.7%)	229 (18.0%)	241 (17.4%)	
Primary or below	251 (9.4%)	124 (9.7%)	127 (9.2%)	
Postgraduate	232 (8.7%)	111 (8.7%)	121 (8.7%)	
Residency registration (hukou)				0.898
Local city	1340 (50.4%)	645 (50.6%)	695 (50.1%)	
Non-local (in-province)	780 (29.3%)	368 (28.9%)	412 (29.7%)	
Non-local (out-of-province)	541 (20.3%)	262 (20.6%)	279 (20.1%)	
Insurance				0.992
URRBMI	1173 (44.1%)	560 (43.9%)	613 (44.3%)	
UEBMI	709 (26.6%)	339 (26.6%)	370 (26.7%)	
Commercial	442 (16.6%)	213 (16.7%)	229 (16.5%)	
None	337 (12.7%)	163 (12.8%)	174 (12.5%)	
Visit type				0.817
New	1332 (50.1%)	635 (49.8%)	697 (50.3%)	
Return	1329 (49.9%)	640 (50.2%)	689 (49.7%)	
Employment schedule				0.984
Daytime	1032 (38.8%)	491 (38.5%)	541 (39.0%)	
Unemployed	445 (16.7%)	218 (17.1%)	227 (16.4%)	
Night	405 (15.2%)	189 (14.8%)	216 (15.6%)	
Rotating	387 (14.5%)	188 (14.7%)	199 (14.4%)	
Evening	261 (9.8%)	127 (10.0%)	134 (9.7%)	
Student	131 (4.9%)	62 (4.9%)	69 (5.0%)	

Continuous variables are median (IQR); categorical variables are no. (%). P values from Mann–Whitney U tests (continuous) or χ² tests (categorical). Abbreviations: URRBMI, Urban–Rural Resident Basic Medical Insurance; UEBMI, Urban Employee Basic Medical Insurance.

**Table 2:** Sleep and clinical characteristics by willingness to enroll.

Characteristic	Total (N = 2661)	Not willing (n=1275)	Willing (n=1386)	P value
Sleep measures				
Sleep duration on workdays, h	6.5 (6.0–7.0)	6.5 (6.0–7.0)	6.5 (6.0–7.0)	0.84
Sleep duration on free days, h	7.5 (7.0–8.0)	7.5 (7.0–8.0)	7.5 (7.0–8.0)	0.877
Sleep latency, min	28.0 (18.0–40.0)	28.0 (18.0–40.0)	28.0 (18.0–40.0)	0.663
Nocturnal awakenings, No./night	2.0 (1.0–3.0)	2.0 (1.0–3.0)	2.0 (1.0–3.0)	0.515
Insomnia Severity Index (0–28)	13.0 (9.0–17.0)	13.0 (9.0–17.0)	13.0 (9.0–17.0)	0.976
Epworth Sleepiness Scale (0–24)	9.0 (7.0–11.0)	9.0 (7.0–11.0)	9.0 (7.0–11.0)	0.858
STOP-Bang score (0–8)	3.0 (2.0–4.0)	3.0 (2.0–4.0)	3.0 (2.0–4.0)	0.852
Symptoms and diagnoses				
Snoring or witnessed apnea, frequency				0.889
Never	906 (34.0%)	433 (34.0%)	473 (34.1%)	
Occasionally	876 (32.9%)	414 (32.5%)	462 (33.3%)	
Frequently	879 (33.0%)	428 (33.5%)	451 (32.5%)	
Previous diagnosis: insomnia	993 (37.3%)	479 (37.6%)	514 (37.1%)	0.801
Previous diagnosis: obstructive sleep apnea	1110 (41.7%)	529 (41.5%)	581 (41.9%)	0.9
Previous diagnosis: depression	939 (35.3%)	453 (35.5%)	486 (35.1%)	0.865
Previous diagnosis: anxiety	1022 (38.4%)	491 (38.5%)	531 (38.3%)	0.938
Chronic pain condition	1258 (47.3%)	605 (47.5%)	653 (47.1%)	0.854
Cardiometabolic disease	1258 (47.3%)	605 (47.5%)	653 (47.1%)	0.854

Continuous variables are median (IQR); categorical variables are no. (%). P values from Mann–Whitney U tests (continuous) or χ² tests (categorical). ISI, Insomnia Severity Index; ESS, Epworth Sleepiness Scale; STOP-Bang, Snoring, Tiredness, Observed apnea, high blood Pressure, BMI, Age, Neck circumference, Gender.

**Table 3:** Knowledge, attitudes, and practices by willingness to enroll.

Characteristic	Total (N = 2661)	Not willing (n=1275)	Willing (n=1386)	P value
Knowledge and attitudes				
Knowledge score (0–6)	4.0 (3.0–5.0)	4.0 (3.0–5.0)	4.0 (3.0–5.0)	0.723
Perceived need for help (1–5)	3.0 (3.0–4.0)	3.0 (3.0–4.0)	3.0 (3.0–4.0)	0.417
Perceived effectiveness: CBT-I vs medication (1–5)	3.0 (3.0–4.0)	3.0 (3.0–4.0)	3.0 (3.0–4.0)	0.27
Trust in sleep specialist (1–5)	4.0 (3.0–4.0)	4.0 (3.0–4.0)	4.0 (3.0–4.0)	0.694
Telehealth acceptability (1–5)	4.0 (3.0–4.0)	4.0 (3.0–4.0)	4.0 (3.0–4.0)	0.772
Privacy concern (1–5)	4.0 (3.0–4.0)	4.0 (3.0–4.0)	4.0 (3.0–4.0)	0.806
Sleep-hygiene adherence (1–5)	3.0 (3.0–4.0)	3.0 (3.0–4.0)	3.0 (3.0–4.0)	0.943
Prior participation in sleep program				0.177
No	2036 (76.5%)	983 (77.1%)	1053 (76.0%)	
Yes, completed	313 (11.8%)	160 (12.5%)	153 (11.0%)	
Yes, not completed	312 (11.7%)	132 (10.4%)	180 (13.0%)	

Continuous variables are median (IQR); categorical variables are no. (%). P values from Mann–Whitney U tests (continuous) or χ² tests (categorical). CBT-I, cognitive behavioral therapy for insomnia.

**Table 4:** Reported barriers to participation.

Characteristic	Total (N = 2661)	Not willing (n=1275)	Willing (n=1386)	P value
Barrier: time (1–5)	3.0 (2.0–4.0)	3.0 (2.0–4.0)	3.0 (2.0–4.0)	0.967
Barrier: cost (1–5)	3.0 (2.0–4.0)	3.0 (2.0–4.0)	3.0 (2.0–4.0)	0.736
Barrier: insurance coverage (1–5)	3.0 (2.0–4.0)	3.0 (2.0–4.0)	3.0 (2.0–4.0)	0.884
Barrier: stigma (1–5)	3.0 (2.0–4.0)	3.0 (2.0–4.0)	3.0 (2.0–4.0)	0.311
Barrier: digital access (1–5)	3.0 (2.0–4.0)	3.0 (2.0–4.0)	3.0 (2.0–4.0)	0.791

Higher scores indicate greater perceived barriers. Continuous variables are median (IQR). P values from Mann–Whitney U tests.

**Table 5:** Univariable logistic regression for willingness to enroll.

Predictor	OR (95% CI)	P value
Demographics		
Sex — Male	1.07 (0.91–1.24)	0.417
Age, y	1.00 (1.00–1.01)	0.491
Travel time to clinic, min	1.00 (1.00–1.00)	0.11
Education		
Education — Junior high	0.98 (0.79–1.22)	0.872
Education — Postgraduate	1.01 (0.68–1.50)	0.967
Education — Primary or below	1.00 (0.77–1.29)	0.987
Education — Senior/vocational	1.01 (0.84–1.21)	0.911
Residence registration (hukou)		
Non-local (in-province)	1.03 (0.86–1.22)	0.778
Non-local (out-of-province)	0.98 (0.80–1.19)	0.829
Insurance		
Commercial	0.99 (0.81–1.21)	0.933
None	0.97 (0.77–1.22)	0.802
URRBMI	1.01 (0.85–1.20)	0.908
Visit and employment		
Visit type — Return	0.98 (0.84–1.14)	0.817
Employment schedule — Evening	0.98 (0.74–1.29)	0.897
Employment schedule — Night	1.05 (0.84–1.30)	0.666
Employment schedule — Rotating	0.99 (0.79–1.24)	0.915
Employment schedule — Student	1.02 (0.71–1.46)	0.915
Knowledge and attitudes		
Knowledge score (0–6)	1.01 (0.96–1.06)	0.723
Perceived need (1–5)	0.97 (0.90–1.04)	0.417
Telehealth acceptability (1–5)	0.99 (0.92–1.06)	0.772
Privacy concern (1–5)	0.99 (0.93–1.06)	0.818
Clinical measures		
ISI (0–28)	1.00 (0.99–1.01)	0.976
ESS (0–24)	1.00 (0.98–1.01)	0.858
STOP-Bang (0–8)	1.00 (0.95–1.06)	0.852
Prior participation		
Prior sleep-program participation — Yes, completed	0.86 (0.67–1.10)	0.226
Prior sleep-program participation — Yes, not completed	1.16 (0.93–1.46)	0.189
Barriers		
Cost (1–5)	0.99 (0.93–1.06)	0.716
Time (1–5)	0.99 (0.93–1.05)	0.781
Stigma (1–5)	0.97 (0.91–1.04)	0.387
Digital Access (1–5)	0.98 (0.92–1.05)	0.606
Insurance (1–5)	0.99 (0.93–1.06)	0.709

Reference categories: Sex (female), Education (college/university), Residence registration (local city), Insurance (UEBMI), Visit type (new), Employment schedule (daytime), Prior sleep program participation (no). Odds ratios are per 1-point increase for continuous predictors unless noted. ISI, Insomnia Severity Index; ESS, Epworth Sleepiness Scale; STOP-Bang, Snoring, Tiredness, Observed apnea, high blood Pressure, BMI, Age, Neck circumference, Gender; URRBMI, Urban–Rural Resident Basic Medical Insurance; UEBMI, Urban Employee Basic Medical Insurance.

**Table 6:** Multivariable logistic regression model A (sociodemographic and risk factors).

Predictor	OR (95% CI)	P value
Demographics		
Sex — Male	1.04 (0.88–1.22)	0.664
Log (1+Travel time), min	0.92 (0.79–1.07)	0.277
Education		
Junior high	1.00 (NE)	
Postgraduate	1.00 (0.67–1.49)	0.993
Primary or below	1.00 (NE)	
Senior/vocational	1.08 (0.89–1.31)	0.449
Residency registration (hukou)		
Non-local (in-province)	1.04 (0.88–1.22)	0.673
Non-local (out-of-province)	0.98 (0.81–1.19)	0.862
Insurance		
Commercial	1.02 (0.84–1.24)	0.862
None	0.98 (0.79–1.23)	0.883
URRBMI	1.03 (0.87–1.22)	0.706
Visit and employment		
Visit type — Return	0.98 (0.85–1.14)	0.803
Employment schedule — Evening	1.02 (0.77–1.35)	0.903
Employment schedule — Night	1.08 (0.86–1.34)	0.488
Employment schedule — Rotating	1.01 (0.81–1.26)	0.939
Employment schedule — Student	1.06 (0.74–1.51)	0.756
Risk flags		
Shift/Night Work	1.05 (0.86–1.28)	0.638
Caregiver	0.96 (0.81–1.14)	0.669
Migrant	0.99 (0.83–1.18)	0.917
Low Socioeconomic Position	0.98 (0.82–1.17)	0.829
Insomnia	0.99 (0.84–1.18)	0.946
OSA Risk	1.02 (0.87–1.19)	0.826
Perinatal Status	0.95 (0.61–1.48)	0.818
Older Age with Multimorbidity	0.97 (0.79–1.19)	0.774
Chronic Pain or Cardiometabolic Disease	0.98 (0.83–1.17)	0.85
Psychological Distress	0.98 (0.83–1.16)	0.824

Model A includes only sociodemographic variables and clinical risk flags. This baseline model serves as comparison for the expanded Model B (**Table 7**) which adds knowledge, attitudes, clinical measures, and barriers. Reference categories: Sex (female), Education (college/university), Residence registration (local city), Insurance (UEBMI), Visit type (new), Employment schedule (daytime). Risk flags are binary indicators (0/1) for high-risk subgroups. OSA, obstructive sleep apnoea; URRBMI, Urban–Rural Resident Basic Medical Insurance; UEBMI, Urban Employee Basic Medical Insurance; NE, not estimable.

**Table 7:** Multivariable logistic regression model.

Predictor	OR (95% CI)	P value
Primary predictors		
Perceived need (per 1-point)	1.20 (1.16–1.25)	<0.001
CBT-I vs medication effectiveness (per 1-point)	1.12 (1.08–1.16)	<0.001
Knowledge score (per 1-point)	1.09 (1.05–1.13)	<0.001
GAD-2 (per 1-point)	1.07 (1.02–1.12)	0.005
Barrier: insurance (per 1-point)	1.05 (1.01–1.09)	0.012
ESS (per 1-point)	1.04 (1.01–1.081)	0.022
Telehealth acceptability (per 1-point)	1.04 (1.001–1.080)	0.046
Age (per 1-year)	1.004 (1.00–1.008)	0.046
Barrier: time (per 1-point)	1.02 (0.981–1.061)	0.323
STOP-Bang (per 1-point)	1.01 (0.966–1.056)	0.66
Trust in sleep specialist (per 1-point)	0.99 (0.951–1.031)	0.626
Barrier: cost (per 1-point)	0.99 (0.950–1.032)	0.633
Barrier: digital access (per 1-point)	0.98 (0.960–1.000)	0.055
Barrier: stigma (per 1-point)	0.97 (0.935–1.007)	0.107
PHQ-2 (per 1-point)	0.94 (0.915–0.966)	<0.001
ISI (per 1-point)	0.93 (0.896–0.966)	<0.001
log(1+Travel time)	0.92 (0.787–1.076)	0.296
Sex		
Female	Reference	
Male	1.02 (0.98–1.19)	0.683
Education		
College/University	Reference	
Senior/vocational	1.10 (0.90–1.33)	0.339
Postgraduate	1.02 (0.68–1.54)	0.924
Residence registration (hukou)		
Local city	Reference	
Non-local (in-province)	1.03 (0.87–1.22)	0.726
Non-local (out-of-province)	0.99 (0.81–1.20)	0.905
Insurance		
UEBMI	Reference	
URRBMI	1.03 (0.87–1.22)	0.734
Commercial	1.04 (0.86–1.27)	0.679
None	0.99 (0.79–1.24)	0.933
Visit type		
New	Reference	
Return	1.00 (0.86–1.17)	0.957
Employment schedule		
Daytime	Reference	
Night	1.06 (0.85–1.33)	0.586
Rotating	1.01 (0.81–1.26)	0.958
Evening	1.02 (0.77–1.36)	0.883
Student	1.06 (0.74–1.52)	0.738
Prior sleep program participation		
No	Reference	
Yes, completed	0.90 (0.70–1.15)	0.405
Yes, not completed	0.94 (0.86–1.03)	0.189

CBT-I, cognitive behavioral therapy for insomnia; ESS, Epworth Sleepiness Scale; ISI, Insomnia Severity Index; GAD-2, Generalized Anxiety Disorder 2-item; PHQ-2, Patient Health Questionnaire 2-item; STOP-Bang, Snoring, Tiredness, Observed apnoea, high blood Pressure, BMI, Age, Neck circumference, Gender; URRBMI, Urban–Rural Resident Basic Medical Insurance; UEBMI, Urban Employee Basic Medical Insurance. All continuous predictors are reported as odds ratios per 1-point increase on the original scale (e.g., Knowledge Score per 1-point increase on 0–6 scale; ISI per 1-point increase on 0–28 scale). For categorical variables, reference categories are: Sex (Female), Education (College/University), Residence registration (Local city), Insurance (UEBMI), Visit type (New), Employment schedule (Daytime), Prior sleep program participation (No).

**Table 8:** Interaction analyses: knowledge by sex and perceived need by psychological distress.

Predictor	OR (95% CI)	P value
Knowledge × Sex		
Knowledge score (per 1-point)	1.07 (0.96–1.19)	0.204
Sex: Male	1.00 (0.76–1.31)	0.992
Knowledge score (per 1-point) × Sex: Male	0.93 (0.82–1.05)	0.229
Perceived need (per 1-point)	0.99 (0.89–1.10)	0.828
Telehealth acceptability (per 1-point)	1.03 (0.93–1.13)	0.592
Perceived Need × Psychological Distress		
Perceived need (per 1-point)	1.07 (0.97–1.18)	0.176
Psychological distress (flag)	1.10 (0.86–1.41)	0.437
Perceived need × Psychological distress	0.93 (0.83–1.06)	0.272
Knowledge score (per 1-point)	1.01 (0.92–1.11)	0.82
Telehealth acceptability (per 1-point)	1.01 (0.92–1.12)	0.801

Interaction models test whether the effect of key predictors varies by subgroups. Model A examines whether knowledge effects differ by sex. Model B examines whether perceived need effects differ by psychological distress status. Continuous variables are per 1-point increase. Reference category for sex is female; reference for psychological distress flag is no distress.

## 3 Results

### 3.1 Demographics, clinical characteristics, and barriers

The study sample comprised 2,661 participants, with 1,386 (52.1%) willing to enroll in a sleep-improvement program and 1,275 (47.9%) not willing. Baseline analysis revealed no significant differences between willing and not willing groups across any demographic variables, including age (median 45 years), sex (56.5% female), education level, residency registration (hukou), insurance type, or employment schedule (all p > 0.05) (**Table 1**). Similarly, sleep measures and clinical characteristics showed no significant differences between groups, with both groups having comparable sleep duration on workdays (median 6.5 hours), sleep duration on free days (7.5 hours), sleep latency (28 minutes), nocturnal awakenings (2 per night), Insomnia Severity Index scores (median 13), Epworth Sleepiness Scale scores (median 9), and STOP-Bang scores (median 3). Previous diagnoses of insomnia (37.3%), obstructive sleep apnea (41.7%), depression (35.3%), and anxiety (38.4%) were also similarly distributed between groups (all p > 0.05) (**Table 2**). Knowledge, attitudes, and practices showed no univariable differences, with both groups reporting similar knowledge scores, perceived need for help, trust in sleep specialists, and telehealth acceptability (all p > 0.05) (**Table 3**). Reported barriers to participation, including time, cost, insurance coverage, stigma, and digital access, also did not differ significantly between groups (**Table 4**). Univariable logistic regression analysis confirmed these findings, with no individual predictor reaching statistical significance (all p > 0.05) (**Table 5**).

### 3.2 Perceived need, CBT-I beliefs, and knowledge emerge as strongest positive predictors

**Figure 2:**
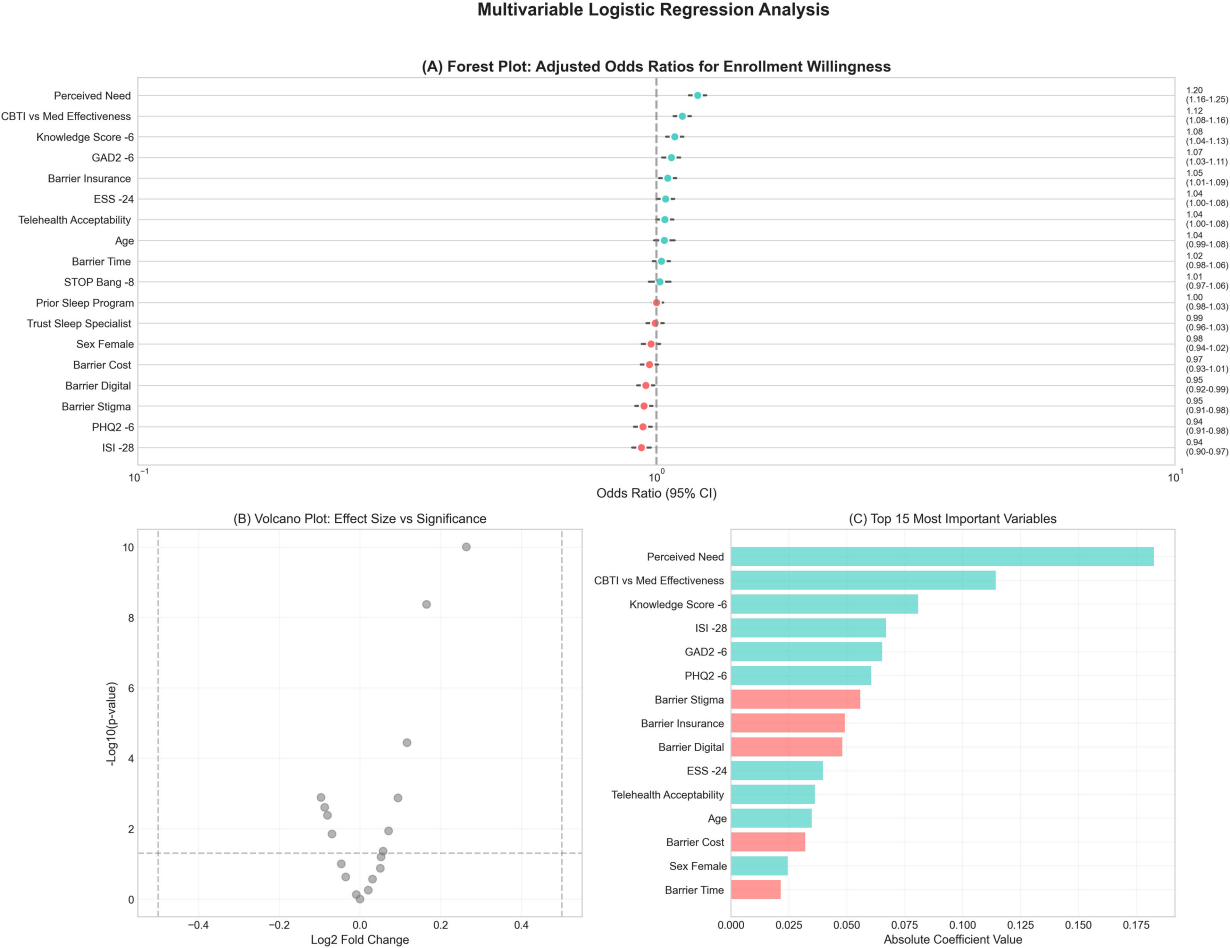
Multivariable logistic regression analysis. Multivariable model identifies cognitive-attitudinal factors as primary predictors. **(A)** Forest plot displays adjusted odds ratios with confidence intervals for all predictors, showing perceived need, CBT-I beliefs, and knowledge as positive predictors (teal), while depression and insomnia severity inversely predict willingness (red). **(B)** Volcano plot illustrates effect size versus statistical significance. **(C)** Bar chart ranks top variables by coefficient magnitude, highlighting dominance of cognitive-attitudinal factors over barriers.

**Figure 3:**
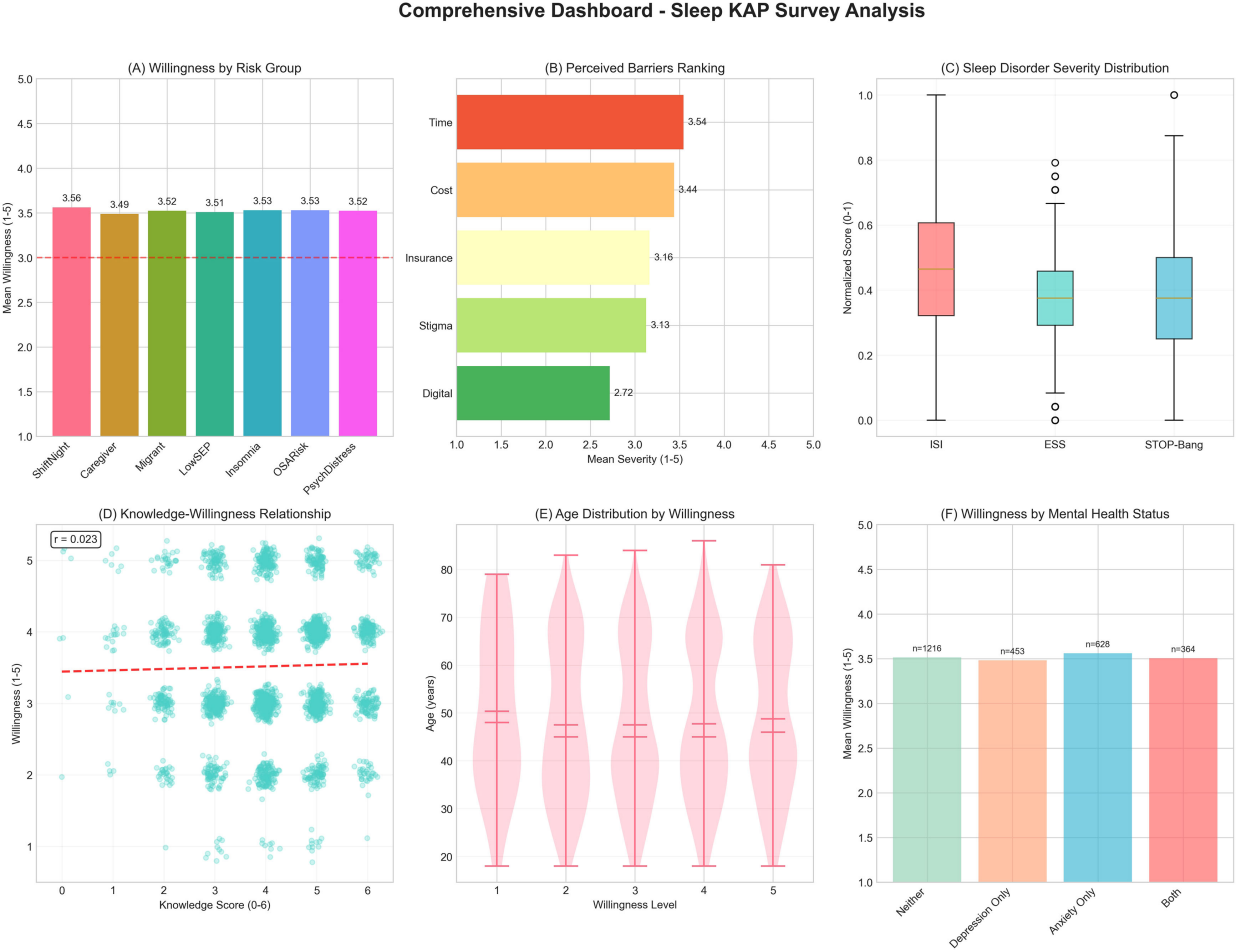
Comprehensive dashboard of sleep KAP survey analysis. Descriptive analyses reveal uniform willingness and weak knowledge-willingness relationship. **(A)** Mean willingness scores across seven at-risk subgroups show minimal variation. **(B)** Perceived barriers ranked by mean severity, with time and cost exceeding social concerns. **(C)** Box plots display distributions of clinical severity measures. **(D)** Scatter plot demonstrates negligible bivariate correlation between knowledge and willingness. **(E)** Violin plots show age distribution across willingness levels. **(F)** Mean willingness by mental health diagnostic groups shows minimal variation.

When controlling multiple variables simultaneously, several factors emerged as significant positive predictors of willingness to enroll in the sleep-improvement program (**Table 7**; **Figure 2A**). Perceived need for help was the strongest predictor (OR = 1.20 per 1-point increase, 95% CI: 1.16–1.25, p<0.001). Over the full scale range (1 to 5), participants with maximum perceived need (score=5) had 2.07-fold higher odds of enrollment willingness compared to those with minimal need (score=1), independent of other factors (**Table 7**; **Figures 2A, C**). Beliefs about CBT-I effectiveness relative to medication also significantly predicted enrollment willingness (OR = 1.12 per 1-point increase, 95% CI: 1.08–1.16, p < 0.001), with participants who viewed cognitive behavioral therapy for insomnia as more effective compared to medication being 12% more likely to be willing to enroll (**Table 7**; **Figures 2A, C**). Knowledge about sleep health was positively associated with willingness (OR = 1.09 per 1-point increase, 95% CI: 1.05–1.13, p < 0.001), with each additional point on the knowledge score (0–6 scale) increasing odds of enrollment by 9% (**Table 7**; **Figures 2A, C**). Anxiety symptoms measured by GAD-2 showed a positive association with willingness (OR = 1.07 per 1-point increase, 95% CI: 1.02–1.12, p = 0.005), suggesting that individuals with higher anxiety were more willing to participate, while daytime sleepiness measured by the Epworth Sleepiness Scale was also positively associated with willingness (OR = 1.04 per 1-point increase, 95% CI: 1.01–1.08, p = 0.022) (**Table 7**; **Figure 2A**). Telehealth acceptability showed a modest positive association (OR = 1.04, 95% CI: 1.00–1.08, p = 0.046), and age showed a modest positive association (OR = 1.004 per year, 95% CI: 1.000–1.008, p=0.046). For each 10-year increase in age, odds of enrollment increased by approximately 4%, suggesting older adults may recognize greater need for sleep intervention (**Table 7**; **Figure 3E**).

### 3.3 Depression and insomnia severity paradoxically associated with reduced willingness to participate

Two clinical measures showed significant inverse associations with enrollment willingness. Depression symptoms (PHQ-2) were associated with 6% lower odds per point increase (OR = 0.94, 95% CI: 0.915–0.966, p<0.001), while insomnia severity (ISI) was associated with 7% lower odds per point increase (OR = 0.93, 95% CI: 0.896–0.966, p<0.001). Thus, a participant with ISI = 21 (severe) versus ISI = 14 (moderate) would have approximately 40% lower odds of willingness, independent of other factors (**Table 7**; **Figure 2A**). Several factors that might have been expected to predict enrollment willingness showed no significant association in the multivariable model, including time barriers (OR = 1.02, p = 0.323), cost barriers (OR = 0.99, p = 0.633), stigma barriers (OR = 0.97, p = 0.107), digital access barriers (OR = 0.98, p = 0.055), trust in sleep specialists (OR = 0.99, p = 0.626), STOP-Bang scores (OR = 1.01, p = 0.66), sex, education level, residency registration, insurance type, visit type, employment schedule, and prior sleep program participation (**Table 7**).

### 3.4 Principal component and cluster analyses for distinct domains of knowledge, clinical, and barrier variables

**Figure 4:**
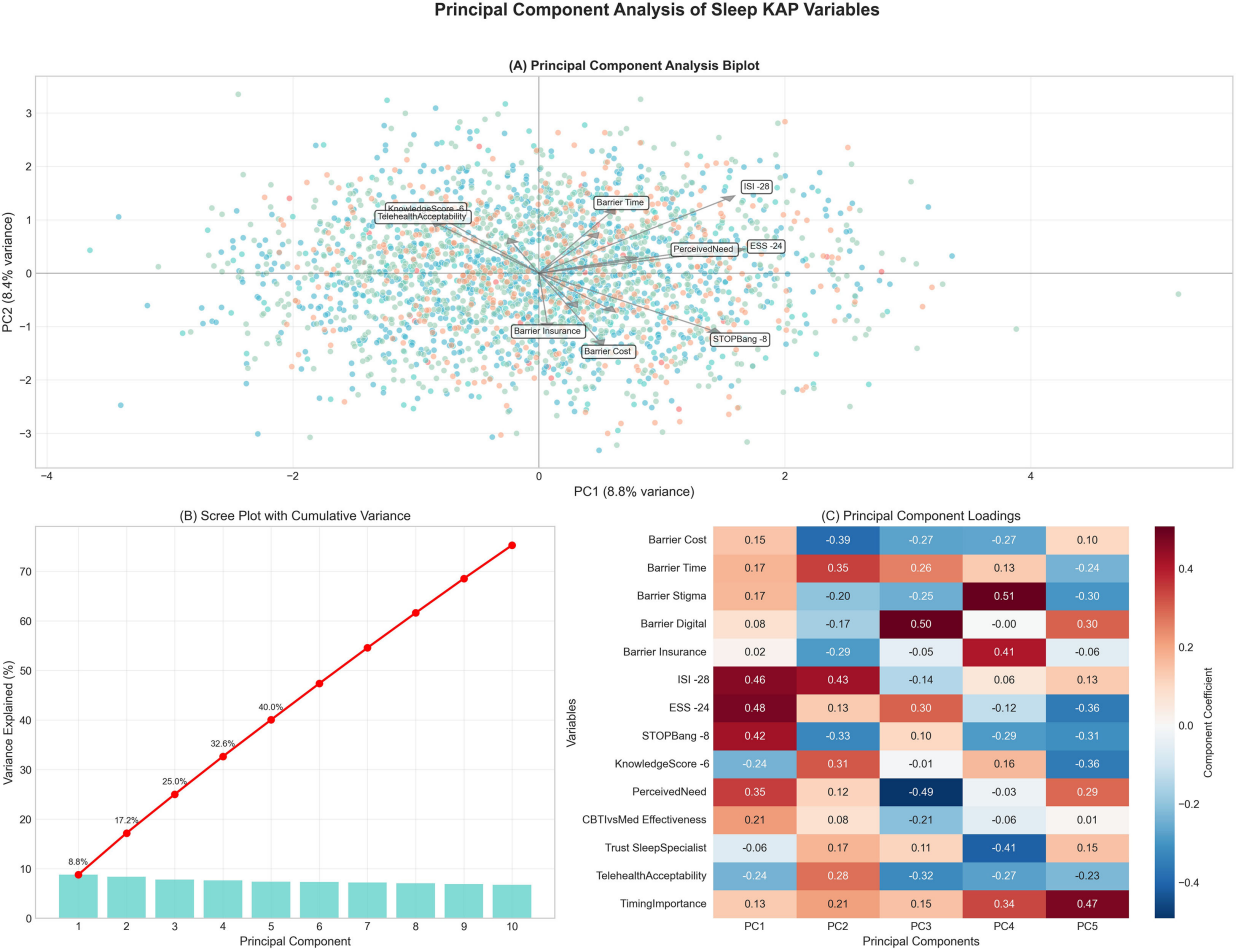
Principal component analysis of sleep KAP variables. Principal component analysis reveals distinct dimensional structure among knowledge-attitudes-practices variables. **(A)** Biplot displays participant distribution across first two principal components with variable loadings shown as arrows. **(B)** Scree plot demonstrates cumulative variance explained by components. **(C)** Heatmap of component loadings shows ISI and perceived need loading strongly on PC1, while barrier variables and knowledge show distinct patterns, confirming separable cognitive-attitudinal, clinical, and barrier domains.

**Figure 5:**
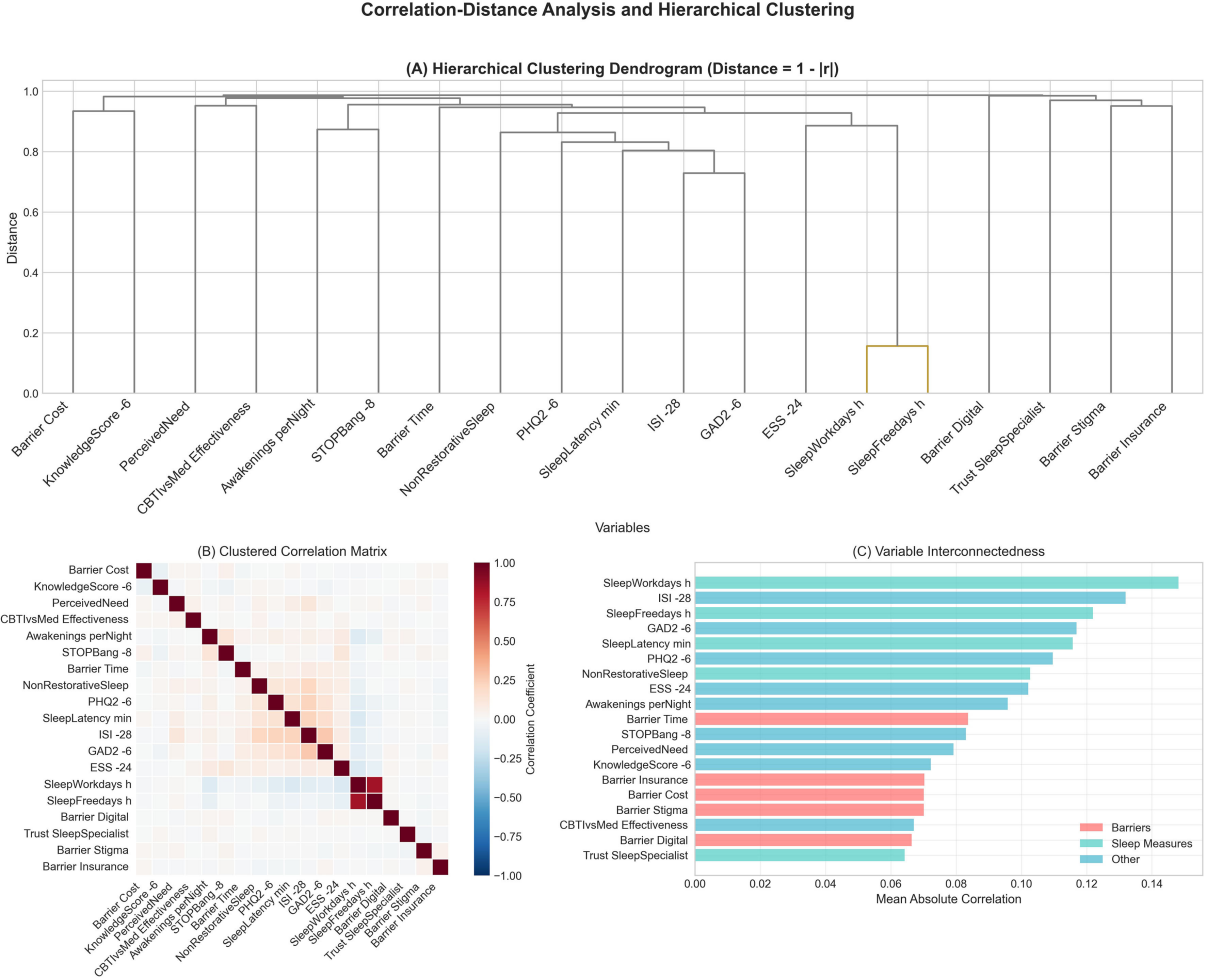
Correlation-distance analysis and hierarchical clustering. Hierarchical clustering identifies three distinct variable domains. **(A)** Dendrogram using correlation distance reveals cognitive-attitudinal cluster, clinical severity cluster, and separate barrier cluster. **(B)** Reordered correlation matrix shows block structure with strong within-cluster and weak between-cluster correlations. **(C)** Variable interconnectedness quantified by mean absolute correlation shows sleep measures most interconnected, while barriers show moderate interconnectedness.

Principal component analysis revealed that the first five components explained 40.6% of the cumulative variance in the Sleep KAP variables, with PC1 accounting for 8.8% and PC2 for 8.4% (**Figure 4B**). The biplot showed that perceived need, barrier time, ISI scores, and ESS scores loaded prominently on the first two principal components, while knowledge score and telehealth acceptability showed distinct loading patterns (**Figures 4A**, **C**). Hierarchical clustering analysis identified distinct groupings of variables, with barrier cost, knowledge score, perceived need, and CBT-I medication effectiveness forming one cluster, suggesting these cognitive and attitudinal factors are interrelated (**Figures 5A**, **B**). Sleep measures including ISI, ESS, STOP-Bang, sleep latency, and nocturnal awakenings formed another coherent cluster, while barrier variables (time, stigma, insurance, digital access) clustered separately, indicating these represent distinct conceptual domains (**Figures 5A**, **B**). Variable interconnectedness analysis showed that sleep duration on workdays had the highest mean absolute correlation with other variables (0.14), followed by ISI scores (0.12) and sleep duration on free days (0.11), while barrier-related variables showed moderate interconnectedness and CBT-I effectiveness beliefs and trust in sleep specialists showed lower correlations with other variables (**Figure 5C**).

### 3.5 Multivariable prediction model shows limited discriminative ability and minimal clinical utility

The final multivariable logistic regression model (Model B) demonstrated modest discriminative ability with an area under the ROC curve (AUC) of 0.543 (95% CI: 0.504–0.590), indicating only slightly better than chance performance in distinguishing willing from not willing participants (**Figure 1A**). At the optimal classification threshold of 0.537 (identified by the Youden index), the overall correct classification rate (accuracy) was 53.8%, with sensitivity 65.1%, specificity 41.5%, PPV 54.8%, and NPV 47.7%. The confusion matrix revealed 271 true positives (correctly predicted willing), 145 true negatives (correctly predicted not willing), 224 false positives (predicted willing but actually not), and 159 false negatives (predicted not willing but actually willing) (**Figure 1B**). Calibration analysis showed reasonable agreement between predicted probabilities and observed frequencies, with slight overestimation at higher probability ranges (**Figure 1C**), while decision curve analysis indicated minimal net benefit from using the model for treatment decisions compared to “treat all” or “treat none” strategies across most threshold probabilities (**Figure 1D**). Interaction analyses tested whether the effects of key predictors varied by subgroups, but neither the interaction between knowledge score and sex (interaction OR = 0.93, 95% CI: 0.82–1.05, p = 0.229) nor the interaction between perceived need and psychological distress (interaction OR = 0.93, 95% CI: 0.83–1.06, p = 0.272) reached statistical significance, indicating that the effects of these predictors were consistent across subgroups (**Table 8**).

### 3.6 Willingness remains uniformly moderate across risk groups with time and cost as primary barriers

Across seven at-risk groups, willingness to participate in sleep health interventions was uniformly moderate, with mean scores on a 5-point scale ranging narrowly from 3.49 to 3.56 (**Figure 3A**). The primary obstacles identified were practical, as time (mean severity 3.54) and cost (3.44) were rated as far more significant barriers than social concerns like stigma (3.13) or technological hurdles like digital access (2.72) (**Figure 3B**). The bivariate correlation between knowledge and willingness was negligible (r=0.023; **Figure 3D**), which initially appears contradictory to the multivariable finding that knowledge independently predicts enrollment (OR = 1.09, p<0.001; **Table 7**). This discrepancy exemplifies confounding: knowledge’s effect is masked in simple correlations but emerges after adjusting for other factors, particularly perceived need and treatment beliefs, with which knowledge is moderately intercorrelated (**Figure 5B**). Similarly, willingness did not meaningfully differ by age, which held a stable median of 45–50 years across all groups (**Figure 3E**), or by mental health status, with scores remaining consistent regardless of an anxiety or depression diagnosis (**Figure 3F**).

## 4 Discussion

This study challenges conventional assumptions about sleep program enrollment. Among 2,661 adults attending a Chinese sleep clinic, we found a striking pattern: standard demographic and clinical factors showed no univariable associations with willingness to enroll, yet multivariable modeling revealed that cognitive-attitudinal factors—perceived need, CBT-I treatment beliefs, and knowledge—were the dominant predictors. Most notably, greater depression and insomnia severity paradoxically decreased enrollment willingness, creating an inverse care law wherein those with greatest need express least readiness to participate. Despite over half of participants (52.1%) expressing willingness to enroll, univariable analyses found no significant demographic, clinical, or barrier-related differences between willing and not willing groups. However, multivariable modeling identified perceived need for help (OR = 1.20), beliefs about CBT-I effectiveness versus medication (OR = 1.12), and sleep health knowledge (OR = 1.09) as the strongest positive predictors of enrollment willingness. Paradoxically, greater depression symptoms (OR = 0.94) and insomnia severity (OR = 0.93) were associated with reduced willingness to participate. Despite identifying significant predictors, the final multivariable model demonstrated limited discriminative ability (AUC = 0.543), suggesting that willingness to participate in sleep improvement programs is a complex phenomenon not adequately captured by traditional knowledge, attitudes, and practices variables alone. Time and cost emerged as the most severe perceived barriers across all risk groups, which showed uniformly moderate willingness to participate.

Our findings align with the Theory of Planned Behavior: proximal beliefs (perceived need, treatment expectancies) outweigh distal attributes (demographics, objective severity) in predicting health behavior. This pattern has been documented in mental health help-seeking more broadly but is newly demonstrated here for sleep programs. The null univariable contrasts likely reflect strong confounding; once perceived need is adjusted for, knowledge and treatment beliefs exert independent effects that bivariate correlations cannot detect (25, 26). Within sleep medicine, qualitative and mixed-methods work has highlighted clinician- and patient-level barriers to identification and referral (e.g., normalization of insomnia, competing medical priorities), such that crude group differences rarely map onto engagement decisions (27, 28). A recent population-based analysis similarly found that individuals attributing sleep disturbance solely to psychological causes were less likely to seek professional help—again, an attitudinal rather than demographic mechanism (29, 30). Our null univariable contrasts therefore cohere with evidence that uptake is primarily cognition- and belief-driven.

Perceived need for help emerged as the strongest independent correlate of willingness, which accords with international evidence that perceived need is a proximal determinant of service use across mental-health conditions (30, 31). In Chinese settings, perceived need similarly predicts help-seeking intention for depression and broader mental-health services (20, 32), supporting the construct’s cross-cultural salience. These convergent findings suggest that case-finding strategies which elevate problem recognition (e.g., brief feedback on symptom scales, risk communication) may meaningfully raise enrollment willingness in sleep programs.

Beliefs that CBT-I is more effective than medication were also independently associated with willingness. This observation is concordant with randomized and observational data demonstrating robust and durable benefits of CBT-I relative to hypnotic pharmacotherapy, including superiority or non-inferiority in head-to-head comparisons and long-term maintenance effects (9, 33, 34). Evidence that CBT-I modifies dysfunctional beliefs about sleep provides a mechanistic rationale for improved patient expectations and subsequent engagement (35). Primary-care surveys indicate that preferences can be split between behavioral and medication approaches, with a substantial proportion favoring CBT-I when informed of comparative effectiveness (34, 36). The present results extend this literature by linking a comparative-effectiveness belief to a concrete enrollment decision in routine clinical flow.

Higher sleep-health knowledge independently predicted willingness, consistent with Chinese and international KAP studies that document associations between knowledge, favorable attitudes, and reported practices in insomnia populations (22, 37). Nevertheless, the bivariate correlation between knowledge and willingness was weak in our data, echoing the well-described intention–behavior gap in help-seeking models (25, 26). Together, these findings imply that knowledge is necessary but insufficient; pairing education with cues to action and logistical enablement may be required to convert insight into enrollment.

Anxiety symptoms were positively associated with willingness, whereas depressive symptoms were inversely associated. Prior research frequently reports that anxiety increases perceived urgency and care-seeking, while depression can reduce motivation through anergia, hopelessness, and heightened self-stigma (38, 39). Behavioral-economic models further posit that depression alters sensitivity to anticipated treatment gains versus costs, diminishing initiation even when need is high (40). Our findings are therefore consistent with a differential motivational signature of common affective symptoms: anxiety appears to mobilize demand, whereas depression dampens it. This asymmetry has practical implications, suggesting that brief activation-enhancing supports (e.g., guided scheduling, micro-incentives) may be required to overcome depressive amotivation during program onboarding.

Greater daytime sleepiness (Epworth Sleepiness Scale) independently predicted willingness, aligning with prior work in sleep clinics where functional impairment—rather than nocturnal symptoms alone—often prompts care-seeking (41). By contrast, greater insomnia severity (Insomnia Severity Index) was inversely associated with willingness after adjustment. Although some studies find that higher severity correlates with care utilisation, evidence is mixed, and several mechanisms could reconcile an inverse association. Severe insomnia frequently co-occurs with depressive symptoms, catastrophising beliefs, and dysfunctional attributions that may promote avoidance or self-reliance (e.g., medication self-management) rather than programme enrollment (35, 42, 43). Additionally, patients with severe, chronic insomnia may have experienced prior treatment failures, producing diminished expectations and lower readiness to engage. Our multivariable models—adjusting for mood symptoms and beliefs—suggest that residual, severity-linked factors (e.g., illness chronicity, prior non-response) could still suppress willingness, an interpretation that merits prospective testing.

Telehealth acceptability was a modest positive correlate of willingness, which is directionally consistent with trials and implementation studies showing that tele-delivered or digital CBT-I is feasible, acceptable, and efficacious across diverse populations, including oncology and older adults (44, 45). In China, a pilot randomized clinical trial of smartphone-based dCBT-I demonstrated clinically meaningful ISI reductions, and national analyses highlight persistent age-related digital divides that could moderate uptake (13, 17). These external data support a policy emphasis on hybrid delivery models that preserve in-person pathways while lowering access frictions for digitally ready patients. Notably, our null univariable contrasts for telehealth acceptability underscore again that simultaneous adjustment is necessary to reveal independent associations.

Two additional findings warrant comment. First, older age showed a small positive association with willingness, which accords with some surveys linking increasing age to greater treatment, stability and higher perceived need for sleep care, although results are heterogeneous across studies and settings (17, 41). Second, perceived insurance barriers showed a modest positive association with willingness (OR = 1.05, 95% CI: 1.01–1.09, p=0.012). Consistent with Andersen’s behavioral model of health service use—wherein perceived need and enabling factors such as coverage and affordability jointly shape care-seeking—this counterintuitive association likely reflects a recognition effect, whereby individuals with stronger enrollment intention become more attuned to financial and insurance constraints rather than deterred by them (46, 47). One plausible explanation is that individuals with higher need and stronger intention become more aware of financial hurdles (a “recognition” effect), such that insurance barriers correlate positively with willingness after adjustment for other factors. Alternatively, insurance concerns may operate as cues to early enquiry in systems where reimbursement is complex. Future qualitative work should unpack whether cost/coverage perceptions are markers of problem recognition or modifiable impediments in this context.

### 4.1 Dimensional structure of determinants

Exploratory principal-component and clustering analyses in the present study suggested separable domains for cognitive/attitudinal variables, clinical severity indices, and barrier items. This pattern mirrors mixed-methods syntheses in insomnia indicating that beliefs and expectancies cluster together and are distinct from symptom severity and logistical constraints (48–50). Such structure reinforces a multi-pronged design logic for interventions: educational components to address misconceptions and build outcome expectations; brief behavioral activation to counter depressive amotivation; and pragmatic facilitation to mitigate time/cost barriers. The modest inter-correlations between trust in specialists and other variables—together with its null association in our models—suggest that trust, while important for adherence, may be less consequential at the initial enrolment decision when compared with perceived need and expected benefit (49, 50).

### 4.2 Model performance and implications for targeting

Despite identifying statistically significant predictors, the multivariable model demonstrated poor discrimination (AUC = 0.543), explaining only ~5% of variance in enrollment decisions (Nagelkerke R²≈0.05). This has three interpretations. First, stated willingness measured on a Likert scale may be an unstable, socially desirable response that does not reflect true behavioral intention. Second, critical determinants are unmeasured, including prior treatment experiences, therapeutic alliance expectations, logistical scheduling constraints, and family/social support. Third, the decision to enroll may be partly stochastic—influenced by momentary motivation, clinician rapport during the encounter, or administrative ease—none of which cross-sectional surveys capture.

These findings underscore inherent limits of questionnaire-based prediction for complex health behaviors. Even comprehensive KAP batteries may miss >90% of behavioral variance. Pragmatically, this suggests enrollment interventions should be offered universally rather than targeting high-risk subgroups, given weak predictive utility. Where resources require prioritization, simple rules (e.g., flag high perceived need + PHQ-2≥3 for motivational interviewing) may be more actionable than probabilistic risk scores. This performance profile is not unusual in behavioral prediction, where intentions and actions are shaped by fluctuating states, unmeasured social context, and dynamic constraints that are poorly captured in cross-sectional surveys (1–3, 37). Methodological guidance for prediction modelling emphasizes that low AUCs should prompt refinement of construct measurement, incorporation of additional behavioral variables (e.g., prior treatment experiences, decisional conflict), and, where feasible, external validation and temporal updating (37–39). Given our findings, pragmatic targeting may be more efficiently anchored to a short battery indexing perceived need, CBT-I comparative belief, knowledge, ESS, and PHQ-2/GAD-2 scores, coupled with simple rules (e.g., flag high perceived need but elevated depression for activation-enhanced onboarding), rather than reliance on probabilistic risk scores.

The Chinese sleep-health landscape is characterized by high population need, evolving specialist capacity, and heterogeneous insurance arrangements (13, 51). Systematic reviews document persistent stigma, low perceived need, and logistical burdens as barriers to professional help-seeking in mental-health domains (20), while digital health expansion is tempered by a widening age-related digital divide (17). Our results are broadly congruent with this context: perceived need and treatment beliefs—proximal cognitive determinants—were paramount; telehealth acceptability modestly facilitated willingness; and time/cost emerged as the most severe barriers in descriptive rankings. The lack of differential effects in risk-flag subgroups and null interactions by sex and psychological distress suggest that these cognitive determinants operate relatively uniformly across common at-risk strata in clinic attendees, supporting generalizability to similar urban outpatient settings. However, this uniformity should be interpreted cautiously in light of the outpatient selection bias described in our limitations: the relative homogeneity of our sample — all clinic-attending, health-seeking adults — may have narrowed the range of cognitive-attitudinal and demographic variation, making differential subgroup effects harder to detect and potentially masking patterns that would emerge in community-based or primary-care cohorts. Nonetheless, replication in rural or lower-resource clinics will be essential, particularly given regional variation in insurance coverage and digital infrastructure.

First, program messaging should foreground the durable, medication-sparing benefits of CBT-I and explicitly link enrolment to personally salient outcomes (e.g., daytime functioning), thereby strengthening perceived benefit expectations (9, 44, 45, 52). Second, brief, scalable interventions to increase perceived need—such as automated feedback from ISI/ESS screeners with tailored risk language—may substantially increase willingness. Third, for patients screening positive for depressive symptoms (PHQ-2), embedding micro-activation supports (e.g., structured scheduling calls or app-based prompts) at the enrolment stage may counter motivational deficits that our data associate with lower willingness (45, 53). Fourth, hybrid delivery with opt-in telehealth can reduce access frictions for digitally ready patients, while maintaining in-person options to avoid exacerbating the digital divide (46, 53). Fifth, transparent, point-of-care information on insurance coverage and costs may convert recognition of financial barriers into feasible action plans; where reimbursement for behavioral therapies is limited, advocacy for coverage parity remains warranted (46, 51, 54).

This study has several strengths, including a large consecutive sample from routine clinical care, comprehensive measurement of knowledge-attitudes-practices constructs integrated with validated clinical severity indices and mental health screeners, prespecified multivariable models testing theoretically grounded predictors, and rigorous attention to model performance evaluation including discrimination, calibration, and clinical decision utility. This study has several important limitations. First, the primary outcome captured stated willingness on a Likert scale rather than observed enrollment behavior; given well-documented intention–behavior gaps across health service domains, prospective designs tracking actual enrollment are required to confirm whether the cognitive-attitudinal predictors identified here translate into genuine behavioral change, and clinical recommendations must therefore remain tentative pending such validation. Second, the outpatient recruitment method introduces several interrelated forms of selection bias that limit internal and external validity. Three distinct mechanisms warrant consideration. First, health-seeking self-selection: all participants had already decided to attend a specialist sleep clinic, indicating a level of illness recognition and motivation for professional care that is absent in community or primary-care samples; willingness proportions reported here therefore almost certainly overestimate those of the broader population with sleep disorders. Second, referral-pathway filtering: many attendees at our tertiary center had been triaged or referred by primary-care physicians or other specialists, meaning the sample is enriched for patients whose presentations were judged clinically significant enough to warrant specialist evaluation, further concentrating individuals with higher perceived need. Third, severity-based exclusion at the point of enrollment: patients with acute medical or psychiatric instability were excluded, selectively removing individuals who may carry the highest symptom burden and, on the basis of our multivariable findings, would be the least willing to enroll — potentially exaggerating the apparent inverse association between depression severity and willingness. Collectively, these mechanisms likely attenuated between-group differences in willingness, inflated the overall willingness proportion relative to a community sample, and may have compressed variance in perceived need and treatment beliefs, reducing our ability to detect the full range of these predictors’ effects. Recruitment from a single tertiary clinic also constrains generalizability to primary care, rural, and lower-resource settings where insurance arrangements, specialist density, and digital infrastructure differ substantially. Third, the modest discriminative ability of the final model (AUC = 0.543) signals that important determinants of enrollment willingness remain unmeasured—including prior treatment experiences, decisional conflict, social support, therapeutic alliance expectations, and program-specific logistical factors—suggesting that cross-sectional KAP batteries, however comprehensive, are structurally insufficient to capture the full complexity of enrollment decision-making. Fourth, the absence of a prospective screening log precludes reporting of a complete participant flow, preventing estimation of non-participation bias and limiting methodological transparency. Fifth, social desirability effects may have inflated knowledge scores and attenuated barrier endorsement; additionally, dichotomizing the willingness scale at ≥4 may have reduced statistical power relative to ordinal or continuous modeling approaches. Finally, the single-country, predominantly urban Chinese clinic context constrains cross-cultural generalizability; replication across diverse health systems—including rural Chinese settings and low- and middle-income countries with differing healthcare financing structures—is required before these cognitive-attitudinal mechanisms can be considered universally applicable. Additionally, the team-constructed KAP items, while demonstrating acceptable internal consistency in the current sample (attitudinal items α = 0.71; barrier items α = 0.68), have not undergone formal confirmatory factor analysis or test-retest reliability assessment in independent samples; their psychometric properties should be further established before adoption in future multi-center or cross-cultural studies.

## 5 Conclusion

Among adults attending a Chinese sleep clinic, cognitive-attitudinal factors—perceived need, CBT-I effectiveness beliefs, and sleep health knowledge—independently predicted willingness to enroll in sleep improvement programs after multivariable adjustment, whereas demographics and practical barriers did not. However, three critical findings qualify these conclusions. First, depression symptoms and insomnia severity paradoxically reduced willingness, creating an inverse care law wherein those with greatest clinical needs were least ready to participate. Second, poor model discrimination (AUC = 0.543) indicates that standard questionnaires capture less than 10% of enrollment variance. Third, this study measured stated willingness rather than actual enrollment behavior; prospective validation linking survey responses to enrollment outcomes is essential before clinical recommendations can be considered definitive. Pending such validation, practical implications include enhancing perceived need through personalized feedback, correcting CBT-I misconceptions, and implementing motivational strategies for patients with depression. Future research should employ prospective designs and test interventions targeting these cognitive determinants to inform evidence-based implementation strategies.

## Data Availability

The raw data supporting the conclusions of this article will be made available by the authors, without undue reservation.

## References

[1] Ramar K Malhotra RK Carden KA Martin JL Abbasi-Feinberg F Aurora RN . Sleep is essential to health: an American Academy of Sleep Medicine position statement. J Clin Sleep Med JCSM Off Publ Am Acad Sleep Med. (2021) 17:2115–9. doi: 10.5664/jcsm.9476 34170250 PMC8494094

[2] Smith MT McCrae CS Cheung J Martin JL Harrod CG Heald JL . Use of actigraphy for the evaluation of sleep disorders and circadian rhythm sleep-wake disorders: an American Academy of Sleep Medicine clinical practice guideline. J Clin Sleep Med JCSM Off Publ Am Acad Sleep Med. (2018) 14:1231–7. doi: 10.5664/jcsm.7230 29991437 PMC6040807

[3] Watson NF Badr MS Belenky G Bliwise DL Buxton OM Buysse D . Recommended amount of sleep for a healthy adult: a joint consensus statement of the American Academy of Sleep Medicine and Sleep Research Society. J Clin Sleep Med JCSM Off Publ Am Acad Sleep Med. (2015) 11:591–2. doi: 10.5664/jcsm.4758 25979105 PMC4442216

[4] Rishi MA Cheng JY Strang AR Sexton-Radek K Ganguly G Licis A . Permanent standard time is the optimal choice for health and safety: an American Academy of Sleep Medicine position statement. J Clin Sleep Med JCSM Off Publ Am Acad Sleep Med. (2024) 20:121–5. doi: 10.5664/jcsm.10898 37904574 PMC10758561

[5] Scott AJ Webb TL Martyn-St James M Rowse G Weich S . Improving sleep quality leads to better mental health: a meta-analysis of randomised controlled trials. Sleep Med Rev. (2021) 60:101556. doi: 10.1016/j.smrv.2021.101556 34607184 PMC8651630

[6] Hertenstein E Feige B Gmeiner T Kienzler C Spiegelhalder K Johann A . Insomnia as a predictor of mental disorders: a systematic review and meta-analysis. Sleep Med Rev. (2019) 43:96–105. doi: 10.1016/j.smrv.2018.10.006 30537570

[7] Yuan K Zheng YB Wang YJ Sun YK Gong YM Huang YT . A systematic review and meta-analysis on prevalence of and risk factors associated with depression, anxiety and insomnia in infectious diseases, including COVID-19: a call to action. Mol Psychiatry. (2022) 27:3214–22. doi: 10.1038/s41380-022-01638-z 35668158 PMC9168354

[8] Dietch JR Blok AC Zhou ES . Improve accessibility to evidence-based treatment for insomnia disorder. Trans Behav Med. (2024) 14:301–3. doi: 10.1093/tbm/ibae006 38402594 PMC11056884

[9] van der Zweerde T Bisdounis L Kyle SD Lancee J van Straten A . Cognitive behavioral therapy for insomnia: a meta-analysis of long-term effects in controlled studies. Sleep Med Rev. (2019) 48:101208. doi: 10.1016/j.smrv.2019.08.002 31491656

[10] Walker J Muench A Perlis ML Vargas I . Cognitive behavioral therapy for insomnia (CBT-I): a primer. Klinicheskaia i Spetsial'naia Psikhologiia = Clin Psychol Special Educ. (2022) 11:123–37. doi: 10.17759/cpse.2022110208 36908717 PMC10002474

[11] Pataka A Kotoulas S Tzinas A Kasnaki N Sourla E Chatzopoulos E . Sleep disorders and mental stress of healthcare workers during the two first waves of COVID-19 pandemic: separate analysis for primary care. Healthcare (Basel). (2022) 10:1395. doi: 10.3390/healthcare10081395 35893217 PMC9394272

[12] Rossman J . Cognitive-behavioral therapy for insomnia: an effective and underutilized treatment for insomnia. Am J Lifestyle Med. (2019) 13:544–7. doi: 10.1177/1559827619867677 31662718 PMC6796223

[13] Zhang C Liu Y Guo X Liu Y Shen Y Ma J . Digital cognitive behavioral therapy for insomnia using a smartphone application in China: a pilot randomized clinical trial. JAMA Netw Open. (2023) 6:e234866. doi: 10.1001/jamanetworkopen.2023.4866 36972049 PMC10043748

[14] Wang J Wu J Liu J Meng Y Li J Zhou P . Prevalence of sleep disturbances and associated factors among Chinese residents: a web-based empirical survey of 2019. J Global Health. (2023) 13:4071. doi: 10.7189/jogh.13.04071 37539543 PMC10401309

[15] Wu W Jiang Y Wang N Zhu M Liu X Jiang F . Sleep quality of Shanghai residents: population-based cross-sectional study. Qual Life Res Int J Qual Life Aspects Treatment Care Rehabil. (2020) 29:1055–64. doi: 10.1007/s11136-019-02371-x 31782018

[16] Espie CA Emsley R Kyle SD Gordon C Drake CL Siriwardena AN . Effect of digital cognitive behavioral therapy for insomnia on health, psychological well-being, and sleep-related quality of life: a randomized clinical trial. JAMA Psychiatry. (2019) 76:21–30. doi: 10.1001/jamapsychiatry.2018.2745 30264137 PMC6583463

[17] Song Y Qian C Pickard S . Age-related digital divide during the COVID-19 pandemic in China. Int J Environ Res Public Health. (2021) 18:11285. doi: 10.3390/ijerph182111285 34769801 PMC8582816

[18] Mahmood A Ray M Ward KD Dobalian A Ahn S . Longitudinal associations between insomnia symptoms and all-cause mortality among middle-aged and older adults: a population-based cohort study. Sleep. (2022) 45. doi: 10.1093/sleep/zsac019 35037061 PMC9189951

[19] Mahmood A Ray M Dobalian A Ward KD Ahn S . Insomnia symptoms and incident heart failure: a population-based cohort study. Eur Heart J. (2021) 42:4169–76. doi: 10.1093/eurheartj/ehab500 34392357 PMC8728724

[20] Shi W Shen Z Wang S Hall BJ . Barriers to professional mental health help-seeking among Chinese adults: a systematic review. Front Psychiatry. (2020) 11:442. doi: 10.3389/fpsyt.2020.00442 32508688 PMC7251144

[21] Freeman S Marston HR Ross C Morgan DJ Wilson G Gates J . Progress towards enhanced access and use of technology during the COVID-19 pandemic: a need to be mindful of the continued digital divide for many rural and northern communities. Healthcare Manage Forum. (2022) 35:286–90. doi: 10.1177/08404704221108314 35855623 PMC9301350

[22] Zhu J Zhang S Zhu Z Wang J Kang T Li X . Knowledge, attitude and practice towards insomnia and sleep hygiene among patients with chronic insomnia in Northwest China in 2023: a cross-sectional survey. BMJ Open. (2024) 14:e083100. doi: 10.1136/bmjopen-2023-083100 38910008 PMC11328641

[23] Shi X Shi Y Wang J Wang H Li Y . Knowledge, attitude, and practice toward sleep disorders and sleep hygiene among perimenopausal women. Sci Rep. (2024) 14:11663. doi: 10.1038/s41598-024-62502-4 38777871 PMC11111451

[24] Wallander MA Johansson S Ruigómez A García Rodríguez LA Jones R . Morbidity associated with sleep disorders in primary care: a longitudinal cohort study. Primary Care Companion to J Clin Psychiatry. (2007) 9:338–45. doi: 10.4088/pcc.v09n0502 17998952 PMC2040284

[25] Doll CM Michel C Rosen M Osman N Schimmelmann BG Schultze-Lutter F . Predictors of help-seeking behaviour in people with mental health problems: a 3-year prospective community study. BMC Psychiatry. (2021) 21:432. doi: 10.1186/s12888-021-03435-4 34479537 PMC8414662

[26] McLaren T Peter LJ Tomczyk S Muehlan H Schomerus G Schmidt S . The Seeking Mental Health Care model: prediction of help-seeking for depressive symptoms by stigma and mental illness representations. BMC Public Health. (2023) 23:69. doi: 10.1186/s12889-022-14937-5 36627597 PMC9831378

[27] Ogeil RP Chakraborty SP Young AC Lubman DI . Clinician and patient barriers to the recognition of insomnia in family practice: a narrative summary of reported literature analysed using the theoretical domains framework. BMC Family Pract. (2020) 21:1. doi: 10.1186/s12875-019-1070-0 31901226 PMC6942394

[28] Koffel E Bramoweth AD Ulmer CS . Increasing access to and utilization of cognitive behavioral therapy for insomnia (CBT-I): a narrative review. J Gen Internal Med. (2018) 33:955–62. doi: 10.1007/s11606-018-4390-1 29619651 PMC5975165

[29] Liu J Ghastine L Um P Rovit E Wu T . Environmental exposures and sleep outcomes: a review of evidence, potential mechanisms, and implications. Environ Res. (2021) 196:110406. doi: 10.1016/j.envres.2020.110406 33130170 PMC8081760

[30] Breslau J North CS Finucane ML Roth E Collins RL . Perceived need for mental health treatment and the mental health response to the COVID-19 pandemic in the United States. Psychiatry. (2022) 85:1–12. doi: 10.1080/00332747.2021.1940470 34328393 PMC8800953

[31] Fisher JH Lichvar E Hogue A Dauber S . Perceived need for treatment and engagement in mental health services among community-referred racial/ethnic minority adolescents. Administration Policy Ment Health. (2018) 45:751–64. doi: 10.1007/s10488-018-0863-0 29525929 PMC6064387

[32] Li XY Liu Q Chen P Ruan J Gong X Luo D . Predictors of professional help-seeking intention toward depression among community-dwelling populations: a structural equation modeling analysis. Front Psychiatry. (2022) 13:801231. doi: 10.3389/fpsyt.2022.801231 35280177 PMC8907597

[33] Lee S Oh JW Park KM Lee S Lee E . Digital cognitive behavioral therapy for insomnia on depression and anxiety: a systematic review and meta-analysis. NPJ Digital Med. (2023) 6:52. doi: 10.1038/s41746-023-00800-3 36966184 PMC10039857

[34] Soh HL Ho RC Ho CS Tam WW . Efficacy of digital cognitive behavioural therapy for insomnia: a meta-analysis of randomised controlled trials. Sleep Med. (2020) 75:315–25. doi: 10.1016/j.sleep.2020.08.020 32950013

[35] Thakral M Von Korff M McCurry SM Morin CM Vitiello MV . Changes in dysfunctional beliefs about sleep after cognitive behavioral therapy for insomnia: a systematic literature review and meta-analysis. Sleep Med Rev. (2020) 49:101230. doi: 10.1016/j.smrv.2019.101230 31816582 PMC7012685

[36] Matteson-Rusby SE Pigeon WR Gehrman P Perlis ML . Why treat insomnia? Primary Care Companion to J Clin Psychiatry. (2010) 12:PCC.08r00743. doi: 10.4088/PCC.08r00743bro 20582296 PMC2882812

[37] An K Wu Z Zhang L Li Y An Z Li S . Knowledge, attitude, and practice of chronic insomnia management among general practitioners in China: a cross-sectional survey. BMC Primary Care. (2024) 25:365. doi: 10.1186/s12875-024-02615-x 39395945 PMC11475601

[38] Tambling RR Russell BS Fendrich M Park CL . Predictors of mental health help-seeking during COVID-19: social support, emotion regulation, and mental health symptoms. J Behav Health Serv Res. (2023) 50:68–79. doi: 10.1007/s11414-022-09796-2 35426011 PMC9009282

[39] Waumans RC Muntingh ADT Draisma S Huijbregts KM van Balkom A Batelaan NM . Barriers and facilitators for treatment-seeking in adults with a depressive or anxiety disorder in a Western-European health care setting: a qualitative study. BMC Psychiatry. (2022) 22:165. doi: 10.1186/s12888-022-03806-5 35247997 PMC8898419

[40] Trusty WT Swift JK Rasmussen EB . A behavioral economic model of help-seeking for depression. Perspect Behav Sci. (2021) 44:541–60. doi: 10.1007/s40614-021-00308-9 35098024 PMC8738822

[41] Chai-Coetzer CL Antic NA McEvoy RD . Identifying and managing sleep disorders in primary care. Lancet Respir Med. (2015) 3:337–9. doi: 10.1016/s2213-2600(15)00141-1 25887981

[42] Morin CM Belleville G Bélanger L Ivers H . The Insomnia Severity Index: psychometric indicators to detect insomnia cases and evaluate treatment response. Sleep. (2011) 34:601–8. doi: 10.1093/sleep/34.5.601 21532953 PMC3079939

[43] Gagnon C Bélanger L Ivers H Morin CM . Validation of the Insomnia Severity Index in primary care. J Am Board Family Med JABFM. (2013) 26:701–10. doi: 10.3122/jabfm.2013.06.130064 24204066

[44] Melton L . Cognitive behavioral therapy for sleep in cancer patients: research, techniques, and individual considerations. J Advanced Practitioner Oncol. (2018) 9:732–40. doi: 10.6004/jadpro.2018.9.7.4 PMC6570518 31249720

[45] Bothelius K Jernelöv S Kaldo V Lu C Stråle MM Jansson-Fröjmark M . Internet-based cognitive behavioural therapy for insomnia comorbid with chronic benign pain - a randomized controlled trial. Internet Interventions. (2024) 38:100781. doi: 10.1016/j.invent.2024.100781 39498476 PMC11533069

[46] Alkhawaldeh A M AL Rayan A Abdalrahim A Musa A Eshah N . Application and use of Andersen's behavioral model as theoretical framework: a systematic literature review from 2012-2021. Iranian J Public Health. (2023) 52:1346–54. doi: 10.18502/ijph.v52i7.13236 37593505 PMC10430393

[47] Hajek A Kretzler B König HH . Determinants of healthcare use based on the Andersen model: a systematic review of longitudinal studies. Healthcare (Basel). (2021) 9:1354. doi: 10.3390/healthcare9101354 34683034 PMC8544403

[48] Bollu PC Kaur H . Sleep medicine: insomnia and sleep. Missouri Med. (2019) 116:68–75. 30862990 PMC6390785

[49] Mei Z Cai C Luo S Zhang Y Lam C Luo S . The efficacy of cognitive behavioral therapy for insomnia in adolescents: a systematic review and meta-analysis of randomized controlled trials. Front Public Health. (2024) 12:1413694. doi: 10.3389/fpubh.2024.1413694 39628800 PMC11613502

[50] Xu D Cardell E Broadley SA Sun J . Efficacy of face-to-face delivered cognitive behavioral therapy in improving health status of patients with insomnia: a meta-analysis. Front Psychiatry. (2021) 12:798453. doi: 10.3389/fpsyt.2021.798453 35002813 PMC8733003

[51] Xu S Li Y Ye J Han D . Sleep medicine in China: current clinical practice. J Clin Sleep Med JCSM Off Publ Am Acad Sleep Med. (2023) 19:2125–31. doi: 10.5664/jcsm.10784 37602465 PMC10692925

[52] Koffel E Amundson E Polusny G Wisdom JP . You're missing out on something great": Patient and provider perspectives on increasing the use of cognitive behavioral therapy for insomnia. Behav Sleep Med. (2020) 18:358–71. doi: 10.1080/15402002.2019.1591958 30907144 PMC6759412

[53] Boerema AM Kleiboer A Beekman AT van Zoonen K Dijkshoorn H Cuijpers P . Determinants of help-seeking behavior in depression: a cross-sectional study. BMC Psychiatry. (2016) 16:78. doi: 10.1186/s12888-016-0790-0 27009062 PMC4806501

[54] Babitsch B Gohl D von Lengerke T . Re-revisiting Andersen's behavioral model of health services use: a systematic review of studies from 1998-2011. Psycho-Social Med. (2012) 9:Doc11. doi: 10.3205/psm000089 23133505 PMC3488807

[55] Awan UA Song Q Ciombor KK Toriola AT Choi J Su T . Demographic and clinicopathologic factors associated with colorectal adenoma recurrence. JAMA Netw Open. (2026) 9 (2):e2556853. doi: 10.1001/jamanetworkopen.2025.568531 41637071 PMC12873766

[56] Awan UA Naeem W Khattak AA Mahmood T Kamran S Khan S . An exploratory study of knowledge, attitudes, and practices toward HPV associated anal cancer among Pakistani population. Front Oncol. (2023) 13:1257401. doi: 10.3389/fonc.2023.1257401 37954070 PMC10637352

[57] Qasim M Awan UA Afzal MS Saqib MAN Siddiqui S Ahmed H . Dataset of knowledge, attitude, practices and psychological implications of healthcare workers in Pakistan during COVID-19 pandemic. Data Brief. (2020) 32:106234. doi: 10.1016/j.dib.2020.106234 32895632 PMC7462453

